# H2A.J is an epigenetic driver of IL-17-associated inflammation in T_H_17 cells and keratinocytes

**DOI:** 10.1186/s43556-026-00611-4

**Published:** 2026-10-08

**Authors:** Tanja Kübelbeck, Anna Stastny, Niklas Beumer, Antonia Kolb, Tanja Knopp, Berenice Fischer, Dennis Fritsche, Ana-Marija Kulis-Mandic, Matthias Klein, Mariia Antipova, Bettina Mros, Michael Delacher, Ari Waisman, Carl Mann, Claudia E. Rübe, Susanne Karbach, Björn E. Clausen, Carsten Deppermann, Miriam Wittmann, Daniela Kramer

**Affiliations:** 1 Department of Dermatology, University Medical Center of the Johannes Gutenberg-University Mainz, Langenbeckstr. 1, 55131 Mainz, Germany; 2 Institute of Immunology, University Medical Center of the Johannes Gutenberg-University Mainz, Langenbeckstr. 1, 55131 Mainz, Germany; 3 Research Center for Immunotherapy, University Medical Center of the Johannes Gutenberg-University Mainz, Langenbeckstr. 1, 55131 Mainz, Germany; 4 Center for Cardiology-Cardiology I, University Medical Center of the Johannes Gutenberg University Mainz, Langenbeckstr. 1, 55131 Mainz, Germany; 5 Center for Thrombosis and Hemostasis, University Medical Center of the Johannes Gutenberg University Mainz, Langenbeckstr. 1, Mainz, 55131 Germany; 6 Institute for Molecular Medicine, University Medical Center of the Johannes Gutenberg-University Mainz, Langenbeckstr. 1, 55131 Mainz, Germany; 7 Université Paris-Saclay, CEA, CNRS, Institute for Integrative Biology of the Cell (I2BC), 91198 Gif-Sur-Yvette, France; 8 Department of Radiation Oncology, Saarland University Medical Center, Kirrberger Str. 100, 66424 Homburg, Saar Germany; 9 German Center for Cardiovascular Research, Partner Site Rhine-Main, Mainz, Germany

**Keywords:** H2A.J, Psoriasis, Keratinocytes, T-cells, IL-17

## Abstract

Psoriasis is a common autoinflammatory disease driven by excessive IL-17 expression. While the pathomechanism of the disease has been revealed, epigenetic factors contributing to psoriasis are less well understood. Here, we investigated the role of the poorly studied histone variant H2A.J in psoriasis. We found that H2A.J gets overexpressed in the skin of psoriasis patients, with even non-lesional skin showing increased expression compared to the skin of healthy donors. To gain mechanistic insights, we studied mice with a global H2A.J knockout (KO) and found them to be partially protected against experimentally induced psoriasis, highlighted by reduced immune cell infiltration and less hyperkeratosis. Molecularly, psoriatic skin of H2A.J KO mice showed diminished expression of chemokines and cytokines, including CXCL1, CCL3, and IL-17A. This phenotype could partially be explained by the role of H2A.J as a key regulator of IL-17A-responsive genes in keratinocytes. Furthermore, we uncovered a T-cell-intrinsic function of H2A.J, which specifically promoted the expression of effector molecules such as *Il17a* and *Il17f* in T_H_17 cells. Our findings from human skin and murine psoriasis models imply that H2A.J acts as a key epigenetic regulator of IL-17-associated inflammation, which controls the (chronic) activation of T_H_17 cells and keratinocytes in psoriasis.

**Supplementary Information:**

The online version contains supplementary material available at https://doi.org/10.1186/s43556-026-00611-4.

## Introduction

Psoriasis combines autoimmune and autoinflammatory features and affects around 4% of the worldwide population [1, 2]. Patients mainly suffer from chronic skin inflammation presenting as scaly, erythematous plaques and comorbidities such as psoriatic arthritis, cardiovascular disease, and metabolic syndrome [3]. The most common subtype of psoriasis is psoriasis vulgaris (plaque psoriasis), which is characterized by excessive skin infiltration of IL-17A-expressing T_H_17 cells. In turn, IL-17A chronically activates keratinocytes, leading to acanthosis, hyperkeratosis, and the induction of several keratinocyte-derived chemokines, such as *CCL20, CXCL8, CXCL1, CXCL2, CCL2*, and *CCL3,* that trigger massive myeloid cell infiltration, prominently neutrophil granulocytes [4]. Accordingly, neutralizing antibodies against IL-17 are highly effective for treatment [3]. Despite therapeutic advances, the causes of psoriasis remain poorly understood, as genetic predispositions are not the leading cause in most cases [5]. Instead, psoriatic skin appears epigenetically predisposed, making individuals hyperresponsive to otherwise harmless insults such as mechanical stress [6]. Which epigenetic aberrations underlie this hypersensitivity remain less well understood.

Despite their high sequence similarity to canonical histones, the regulation of expression of the more than 17 known replication-independent H2A variants remains largely unknown [7]. Specific chaperone complexes replace classical histones with histone variants at specific promoter and enhancer regions, affecting promoter accessibility and recruitment of chromatin remodelers and epigenetic factors, ultimately determining gene expression [7, 8]. Thus, histone variants contribute to fundamental cell processes, including chromosomal integrity, DNA damage response, and cell fate transition, and global deletion of some variants results in embryonic lethality [9, 10].

The histone variant H2A.J (encoded by *H2AJ or H2afj*) differs from canonical H2A by a valine-for-alanine substitution at position 11 and a unique phosphorylatable SQ motif at the C-terminus [11]. Previous studies have shown that H2A.J accumulates in senescent fibroblasts following permanent DNA damage, and in aged skin due to altered differentiation and chromatin remodeling [12, 13]. Consequently, H2A.J promoted the expression of senescence-associated secretory phenotype (SASP)-associated cytokines and chemokines, such as *CXCL1*, *CXCL10*, and *IL1B* [11, 12]. Loss of H2A.J in skin exaggerated radiation-induced dermatitis and subsequent tissue damage by regulating inflammatory mediators, including myeloid-recruiting chemokines [14].

Given that H2A.J target genes include cytokines and chemokines central to psoriasis-associated inflammation, we investigated the role of H2A.J in psoriasis. We found H2A.J to be highly expressed in non-lesional and lesional psoriatic skin compared to healthy skin, and this increased expression was especially detectable in keratinocytes, T-cells, and dendritic cells (DC). Global *H2afj* deletion attenuated experimentally induced psoriasis-like skin inflammation in mice by diminishing keratinocyte activation and impairing the recruitment of myeloid cells and IL-17A-expressing T-cells. It also affected systemic inflammation, potentially by suppressing T_H_17 differentiation and activation. Subsequently, H2A.J promoted effector molecule expression, especially in activated keratinocytes and T_H_17 cells. Thus, we identified excessive H2A.J expression as a key epigenetic mechanism driving IL-17A-related inflammation in psoriasis by affecting the activation and function of both T_H_17 cells and keratinocytes.

## Results

### The histone variant H2A.J is selectively overexpressed in the skin of psoriasis patients

**Fig. 1:**
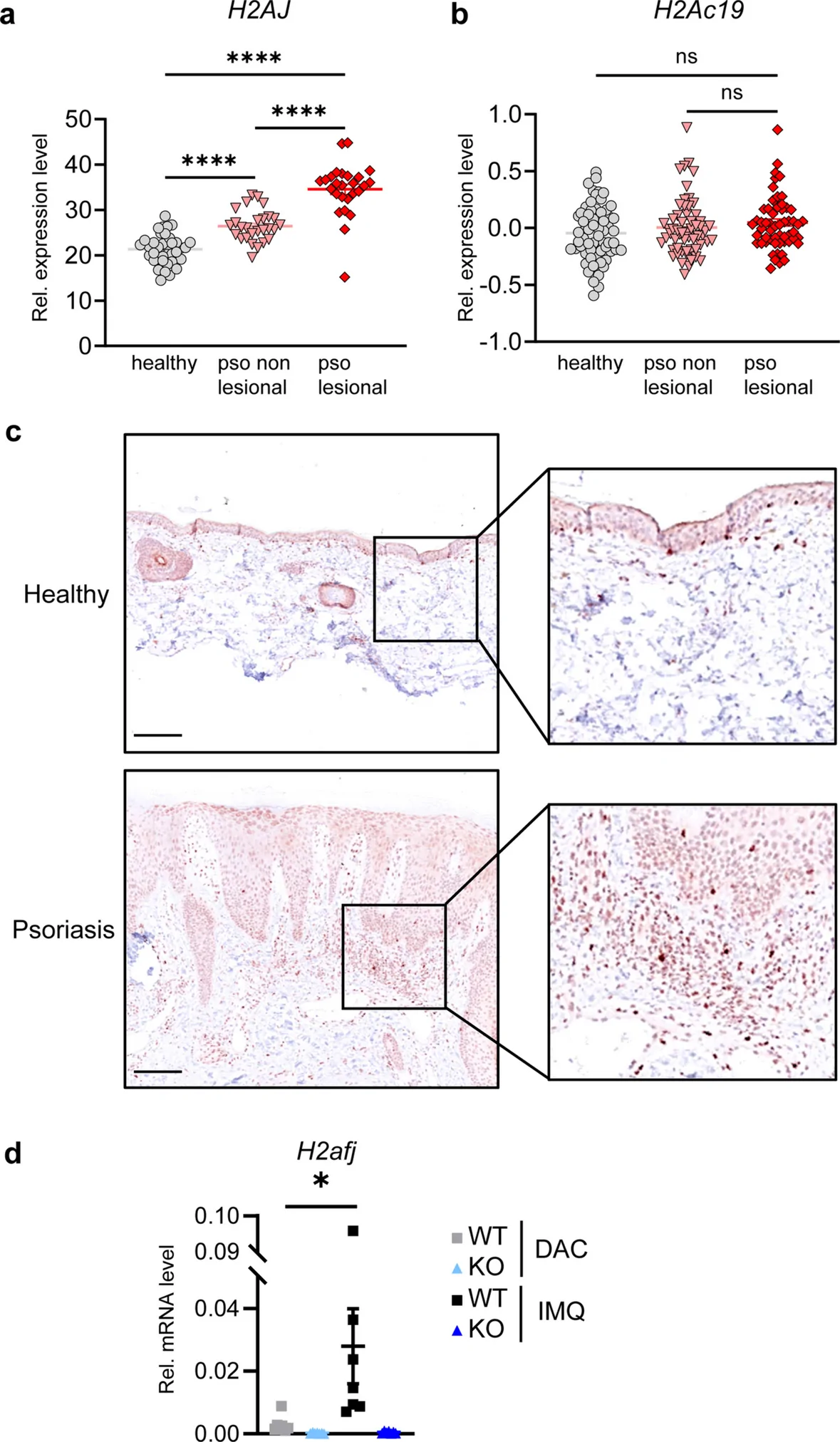
The histone variant H2A.J is specifically overexpressed in the skin of psoriasis patients. **a** + **b** Gene expression analysis from samples derived from healthy skin and from non-lesional and lesional skin of psoriasis patients. Shown is the mean of the relative number of reads per gene derived from bulk RNA sequencing, after normalization to the total amount of reads per sample ± SEM. The data were retrieved from a previously published dataset [15, 16]. **a** *H2AJ* mRNA levels (*n* = 37 healthy skin samples), (*n* = 27 non-lesional skin of psoriasis patients), (*n* = 26 lesional skin of psoriasis patients). **b** *H2AC19* mRNA levels and *H2AJ* mRNA levels (*n* = 64 healthy skin samples, *n* = 58 non-lesional and lesional skin of psoriasis patients). **c** Immunohistochemical staining of H2A.J in skin samples from healthy donors and lesional skin from psoriasis patients. Scale: 200 μm.** d** Gene expression analysis of the skin of DAC (Base gel emulsifying hydrophobic ointment) as vehicle control or IMQ-treated WT and H2A.J KO mice at day 6. Relative *H2afj* mRNA levels were normalized to the reference gene *Actb*. Shown is the mean of 7 biological replicates ± SEM. Significance was calculated using Brown-Forsythe and Welch ANOVA multiple comparison test (**a** + **b**) and a 2-tailed Student’s t-test (**d**) (*p < 0.05, ****p < 0.0001, *ns* = not significant)

A previous study identified H2A.J as a key regulator of the so-called senescence-associated secretory phenotype (SASP) [11]. We became interested in studying H2A.J in the context of psoriasis, as the expression of multiple chemokines and cytokines, which are also known to promote psoriasis-associated inflammation, was crucially dependent on the presence of H2A.J, at least in senescent fibroblasts [11, 12]. Consequently, we investigated the expression pattern of H2A.J (encoded by *H2AJ or H2afj*) in human skin biopsies derived from healthy donors without any inflammatory skin disease, lesional and non-lesional psoriatic skin of patients with plaque psoriasis (Fig. 1a-c). Using a published dataset [15, 16], we revealed that *H2AJ* mRNA levels were already increased in the non-lesional skin of psoriasis patients compared to healthy skin and further increased in psoriatic lesions (Fig. 1a). This was not due to a general upregulation of H2A mRNA, as the mRNA levels of a classical H2A gene (*H2AC19*) remained similarly expressed in lesional, non-lesional, and healthy skin (Fig. 1b). Instead, we specifically detected increased H2A.J protein expression in both the epidermis and the dermis of psoriatic skin (Fig. 1c). Furthermore, psoriasis-like skin inflammation induced by the topical treatment with the TLR7/8 agonist Imiquimod (IMQ) led to increased *H2afj* expression in the skin of wild-type (WT) mice (Fig. 1d). Thus, we could show that H2A.J expression is significantly enhanced in human psoriatic skin and murine psoriasis-like skin inflammation.

### A global knockout of H2A.J partially protects against experimentally induced psoriasis in mice

**Fig. 2:**
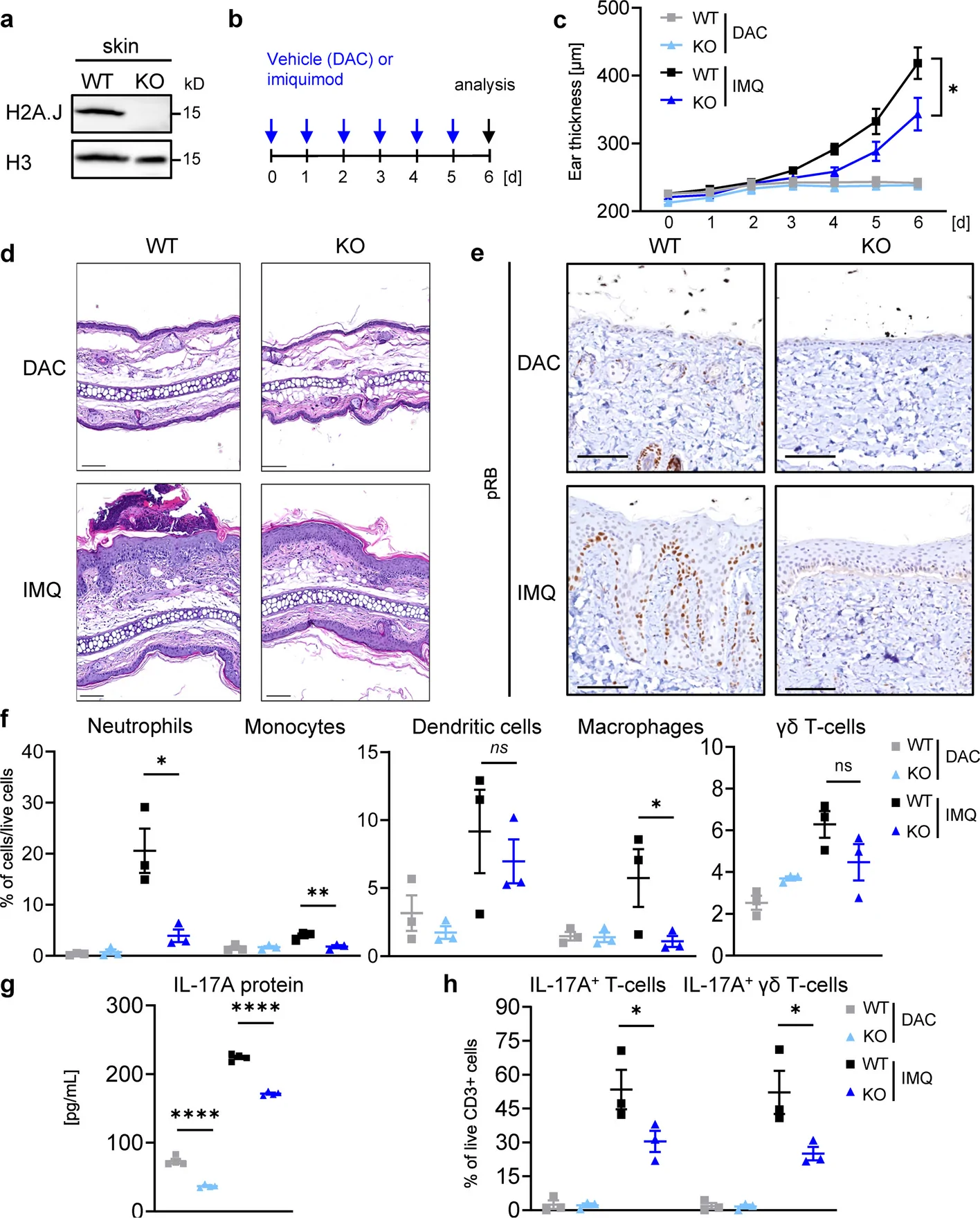
A global knockout of H2A.J partially protects against experimentally induced psoriasis in mice. **a** Immunoblot confirming H2AJ KO in the skin of global H2AJ KO mice. H3 served as a loading control. **b** Treatment scheme. **c** Ear thickness during the course of the experiment. Shown is the mean of 6 biological replicates ± standard error (SEM). **d** H&E staining of ears from WT (wild-type) and H2A.J KO mice treated with DAC cream or IMQ at day 6. Scale: 100 μm. **e** Immunohistochemical staining of pRB (phosphorylated retinoblastoma protein) in skin samples upon treatment with DAC or IMQ at day 6. Scale: 100 μm. **f** Flow cytometry analysis of skin-infiltrating immune cells at day 6 upon DAC or IMQ treatment. Shown is the percentage of viable, positive cells. The following stainings were applied: neutrophils: Ly6C^+^ Ly6G^+^, monocytes: Ly6G^−^, Ly6C^+^, DC: CD11c^+^, macrophages: F4/80^+^, γδ T-cells: γδTCR^+^, Depicted is the average of 3 biological replicates ± standard error (SEM). **g** IL-17A enzyme-linked immunosorbent assay from skin of DAC- or IMQ-treated WT and H2A.J KO mice. Shown is the mean of 4 biological replicates ± SEM. **h** Assessment of IL-17A-producing T-cells in DAC- and IMQ-treated back skin of WT and H2A.J KO mice at day 6 by flow cytometry. After pre-gating on viable, CD3^+^ cells, cells were gated on IL-17A expression alone or on γδTCR^+^ and IL-17A.^+^. Shown is the mean of 3 biological replicates ± SEM. Significance was calculated using a 2-tailed Student’s t-test (*p < 0.05, **p < 0.01, ***p < 0.001, ****p < 0.0001, *ns* = not significant)

Based on our findings in human patients, we wanted to further investigate the consequences of H2A.J expression in psoriasis. For this purpose, we analyzed the effect of a global H2A.J knockout (KO) in mice, compared to wild-type control mice (WT) derived from backcrosses of H2A.J KO mice, to avoid inbred effects. As reported before, global H2A.J KO mice were viable, fertile, and did not show any obvious skin phenotype at steady state [14, 17] (Fig. S1a and b). Using these two mouse strains, we topically treated the ears and back skin with DAC (Base gel emulsifying hydrophobic ointment) as vehicle control or IMQ for 6 consecutive days (Fig. 2a and b), and analyzed the mice on day 6, which represents a well-defined model for the investigation of psoriasis-like skin inflammation in mice [18].

Upon IMQ treatment, global H2A.J KO mice displayed significantly less ear swelling (Fig. 2c and d, Fig. S1c) and a reduced PASI (Psoriasis Associated Disease Index) score (Fig. S1d), both markers for inflammation severity, compared to IMQ-treated wild-type control mice. Furthermore, histological analysis of the IMQ-treated ears and back skin revealed less microabscess formation, reduced immune cell infiltration, and overall, less keratinocyte hyperproliferation and acanthosis in H2A.J KO mice (Fig. S1e and f). This was also validated by staining for phosphorylated Retinoblastoma protein (pRB), a marker of cell proliferation, which showed far less hyperkeratosis in the back skin of IMQ-treated H2A.J KO mice compared with IMQ-treated control mice (Fig. 2e). Finally, we investigated immune cell infiltration of DAC control cream- and IMQ-treated back skin by flow cytometry. Whereas IMQ-treated wild-type control mice displayed increased skin infiltration of neutrophils, monocytes, and macrophages at day 6 of IMQ treatment, IMQ-treated H2A.J KO mice had significantly less infiltration of these immune cell types into the IMQ-challenged back skin (Fig. 2f, Fig.S1g). Importantly, the total protein levels of the key cytokine IL-17A (Fig. 2g), as well as the number of IL-17A-expressing CD3^+^ and γδ^+^ T-cells, were also significantly reduced in the back skin of IMQ-treated global H2A.J KO mice (Fig. 2h). Thus, global deletion of H2A.J alleviates the development of IMQ-induced psoriasis-like skin inflammation in mice by affecting keratinocyte hyperproliferation and the recruitment of a range of immune cell subsets into the affected skin areas.

### H2A.J deletion results in impaired expression of psoriasis-associated chemokines and cytokines in the skin of IMQ-treated mice

**Fig. 3:**
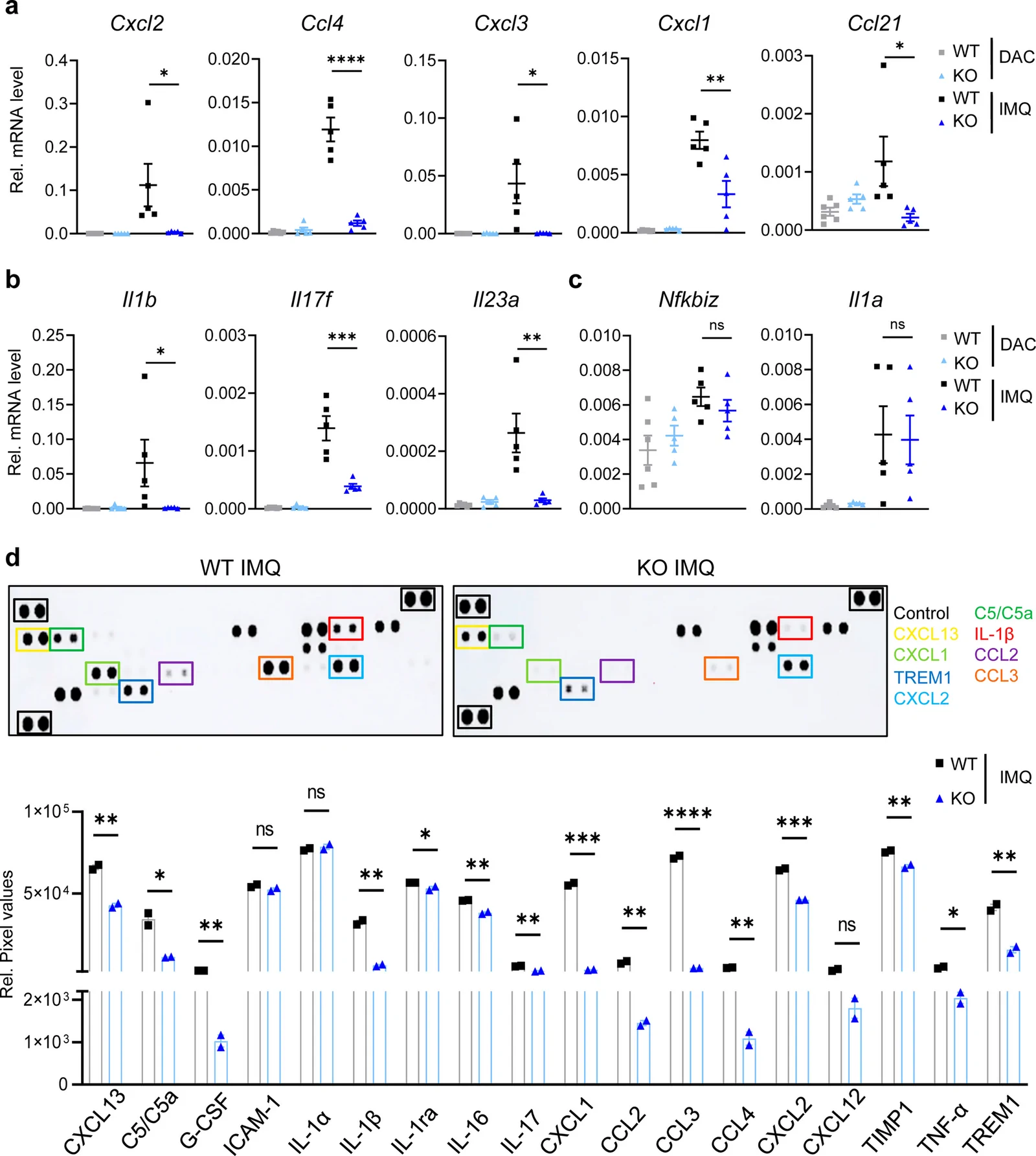
H2A.J promotes the expression of several cytokines and chemokines in the IMQ-treated skin.** a**-**c** RT-qPCR of the skin of DAC or IMQ-treated WT and H2A.J KO mice at day 6. Relative mRNA levels were normalized to the reference gene *Actb.* Shown is the mean of 5 or 6 (WT DAC) biological replicates ± SEM. **a** mRNA levels of different chemokines. **b** Gene expression levels of several psoriasis-associated cytokines. **c** IMQ-induced genes that H2A.J. does not regulate. **d** Protein levels of various chemokines and cytokines from the skin of WT and H2A.J KO mice treated with IMQ. Shown is the immunoblot analysis and the evaluation normalized to the reference spots as relative pixel values. For each membrane, lysates derived from the skin of three mice were pooled in equal amounts and analyzed. Shown is the mean ± SEM. Significance was calculated using a 2-tailed Student’s t-test (*p < 0.05, **p < 0.01, ***p < 0.001, ****p < 0.0001, *ns* = not significant)

As the deletion of H2A.J partially suppressed the development of experimentally induced psoriasis in vivo, we subsequently investigated which subsets of cytokines and chemokines are affected by the loss of H2A.J. Gene expression analysis of whole skin biopsies revealed the complete absence of multiple neutrophil and monocyte-attracting chemokines, such as *Cxcl1-3*, *Ccl4*, and *Ccl21*, in IMQ-treated mice lacking H2A.J., which possibly explains the observed, significant reduction of neutrophil, monocyte, and macrophage recruitment into the challenged skin areas of these mice (Fig. 3a). Furthermore, multiple cytokines known to promote keratinocyte and immune cell activation in psoriasis, such as *Il1b*, *Il17f*, and *Il23a*, were strongly reduced in IMQ-treated skin lacking H2A.J, thereby likely contributing to the observed suppression of hyperkeratosis (Fig. 3b). By contrast, other known inflammatory mediators that promote skin inflammation, such as *Nfkbiz* or *Il1a*, were equally induced in IMQ-treated skin of both mouse strains, thus implying an H2A.J-dependent transcriptional control of only a subset of psoriasis-associated genes (Fig. 3c). Importantly, however, these effects also translated to the protein level, as similar cytokines and chemokines, including IL-1β, CXCL1, and CXCL2, as well as IL-17, potentially derived from T-cells, were strongly reduced in the skin of IMQ-treated H2A.J KO mice (Fig. 3d).

Thus, our findings highlight that H2A.J is needed to fully trigger IMQ-induced psoriasis-like skin inflammation in mice, as it is crucial for the expression of a subset of chemokines and cytokines, leading to enhanced neutrophil, monocyte, and T_H_17 cell recruitment and, subsequently, promoting inflammation.

### Global knockout of H2A.J attenuates IL-17A- and IL-36-mediated psoriasis-like skin inflammation

**Fig. 4:**
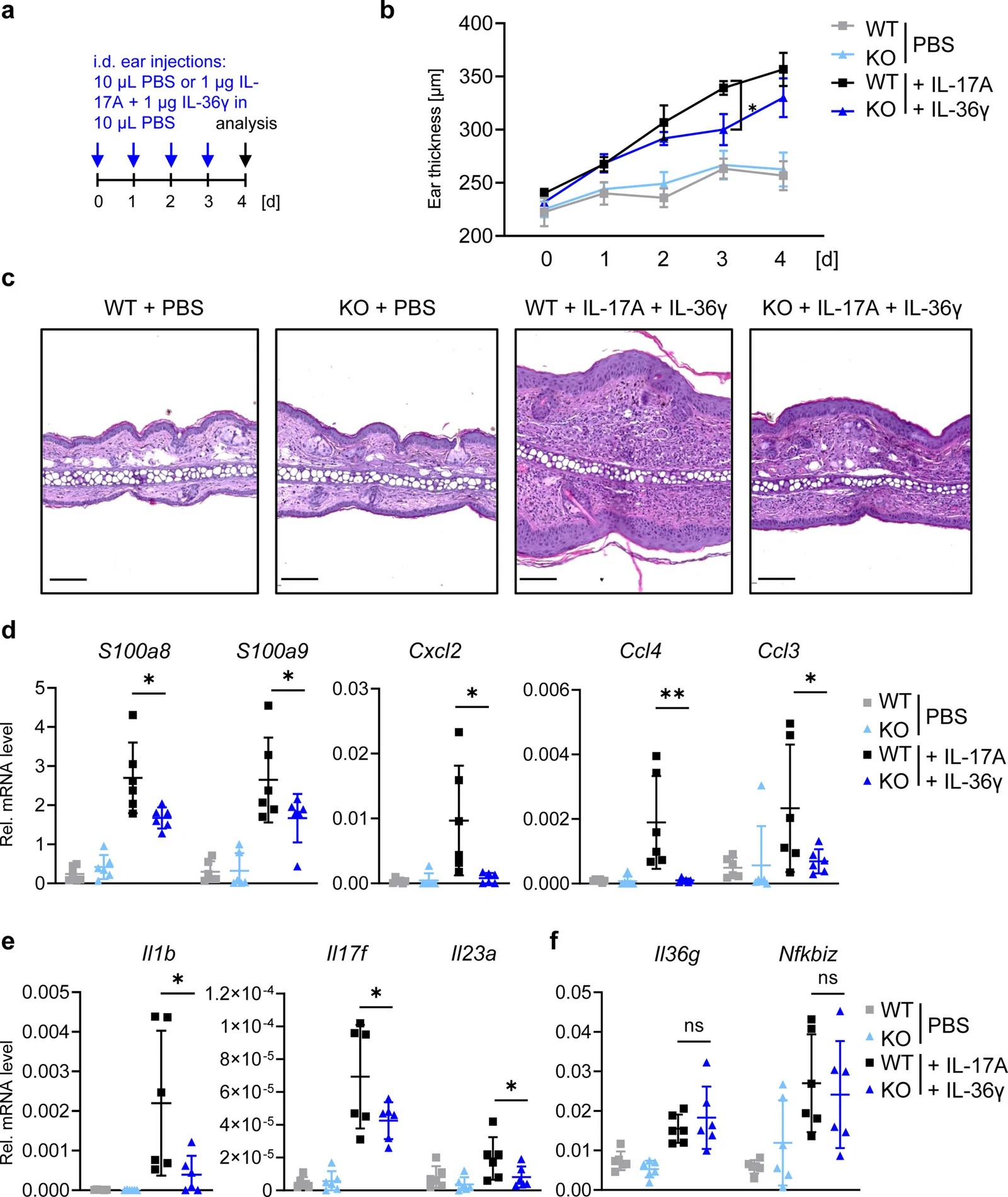
IL-17A- and IL-36γ-mediated psoriasis-like skin inflammation is attenuated by a global H2A.J knockout.** a** Treatment scheme. **b** Ear thickness during the course of the experiment. Shown is the mean of 6 biological replicates ± standard error (SEM). **c** H&E staining of ears from WT and H2A.J KO mice injected with PBS or IL-17A and IL-36γ at day 4. Scale: 100 μm. **d**-**f** RT-qPCR of the ears of WT and H2A.J KO mice injected with PBS or IL-17A and IL-36γ at day 4. Relative mRNA levels were normalized to the reference gene *Rpl37a.* Shown is the mean of 6 biological replicates ± SEM. Shown is the mean ± SEM. Significance was calculated using a 2-tailed Student’s t-test (*p < 0.05, **p < 0.01, *ns* = not significant)

Though IMQ is a widely used and accepted model for analyzing psoriasis-like skin inflammation in mice, some reports have also highlighted its limitations in its comparability to human psoriasis pathogenesis [19, 20]. An attractive alternative model for studying psoriasis in mice is intradermal injections of recombinant IL-17A and biologically active IL-36γ [21–23]. We consequently analyzed the effect of a global H2A.J deletion on IL-17A- and IL-36γ-induced skin dermatitis in mice. For this purpose, mice received daily intradermal injections of both cytokines into the ears of wild-type and H2A.J KO mice for 4 days (Fig. 4a). In parallel, control mice received intradermal injections of PBS. Whereas IL-17A- and IL-36γ-treated wild-type mice displayed increased ear swelling and hyperkeratosis, compared to PBS-treated control mice, the overall inflammation was attenuated in IL-17A- and IL-36γ-treated global H2A.J KO mice (Fig. 4b and c, Fig. S2a). This was also reflected in a decrease in infiltrating MPO-positive cells in treated KO skin, indicating fewer infiltrating monocytes and neutrophils (Fig. S2b). Additionally, a slightly attenuated pRB staining was observed in the IL-17A- and IL-36γ-treated H2A.J KO ears, thereby implicating less severe hyperkeratosis (Fig. S2c). Consistently, a subset of psoriasis-associated key cytokines and chemokines was significantly less expressed in the ears of IL-17A- and IL-36γ-treated global H2A.J KO mice upon 4 days of treatment, compared to the treated wild-type mice (Fig. 4d-f). Thus, H2A.J not only promotes IMQ-mediated, but also IL-17A- and IL-36γ-dependent psoriasis-like skin inflammation in mice.

### IMQ-treated, global H2A.J KO mice show alleviated systemic inflammation

**Fig. 5:**
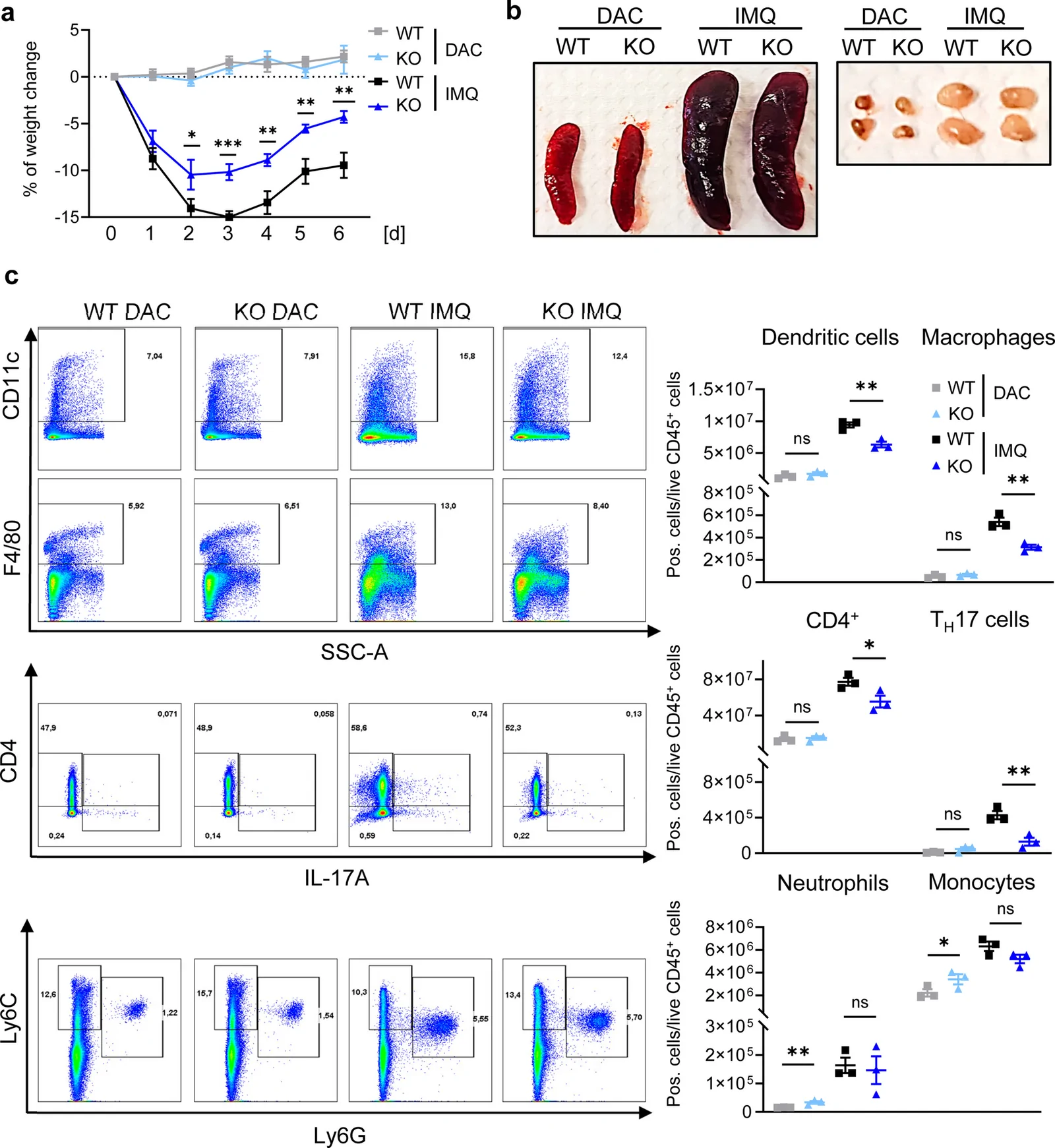
Global H2A.J KO mice exhibit reduced systemic inflammation following IMQ treatment.** a** Percentage of the body weight change during the course of topical DAC or IMQ treatment. Data derived from the mean of 8 biological replicates per group ± SEM. **b** Pictures of spleen and inguinal lymph nodes of DAC- or IMQ-treated WT and H2A.J KO mice at day 6. **c** Flow cytometry analysis of the relative percentage of immune cells in the spleen of DAC- or IMQ-treated WT and H2A.J KO mice at day 6. Shown is the number of positive cells of 3 biological replicates ± SEM, after pre-gating on viable CD45^+^ cells. The following staining’s were applied: DC: CD11c^+^, macrophages: F4/80 +, T-cells: CD4^+^, T_H_17 cells CD4^+^ IL-17A^+^, neutrophils: Ly6C^+^ Ly6G^+^, monocytes Ly6G^−^, Ly6C.^+^. Significance was calculated using a 2-tailed Student’s t-test (*p < 0.05, **p < 0.01, ***p < 0.001, ns = not significant)

Topically applied IMQ also induces systemic inflammation in mice, associated with body weight loss, increased myelopoiesis, and splenomegaly [24, 25]. We therefore examined whether H2A.J knockout also affected systemic inflammation, possibly due to a general intrinsic role of H2A.J in the affected immune cell subsets. Interestingly, IMQ-treated wild-type mice suffered from significantly more body weight loss than IMQ-treated H2A.J KO mice (Fig. 5a, Fig. S3a), whereas the IMQ treatment led to similarly enlarged spleens and lymph nodes in both experimental groups (Fig. 5b). Importantly, IMQ-treated wild-type mice displayed increased relative numbers of DC, macrophages, and IL-17A-producing CD4^+^ T-cells in the spleen, but significantly lower amounts of the respective cell types could be detected in IMQ-treated H2A.J KO mice (Fig. 5c). By contrast, similar numbers of neutrophils and monocytes could be detected in the spleen and bone marrow of IMQ-treated wild-type and H2A.J KO mice (Fig. 5c and Fig. S3b). We conclude from these datasets on the one hand that the generation of monocytes and neutrophils, as well as their tissue infiltration into the spleen, remain unperturbed. On the other hand, impaired recruitment of neutrophils and monocytes into the skin of IMQ-treated H2A.J KO mice is likely due to the absence of keratinocyte-derived chemotactic factors. However, a significant reduction of DC and T_H_17 cells in the skin and spleen of IMQ-treated H2A.J KO mice could imply that H2A.J might be intrinsically needed in both cell types to regulate their differentiation and activation.

### Cell-type-specific expression of H2A.J

**Fig. 6:**
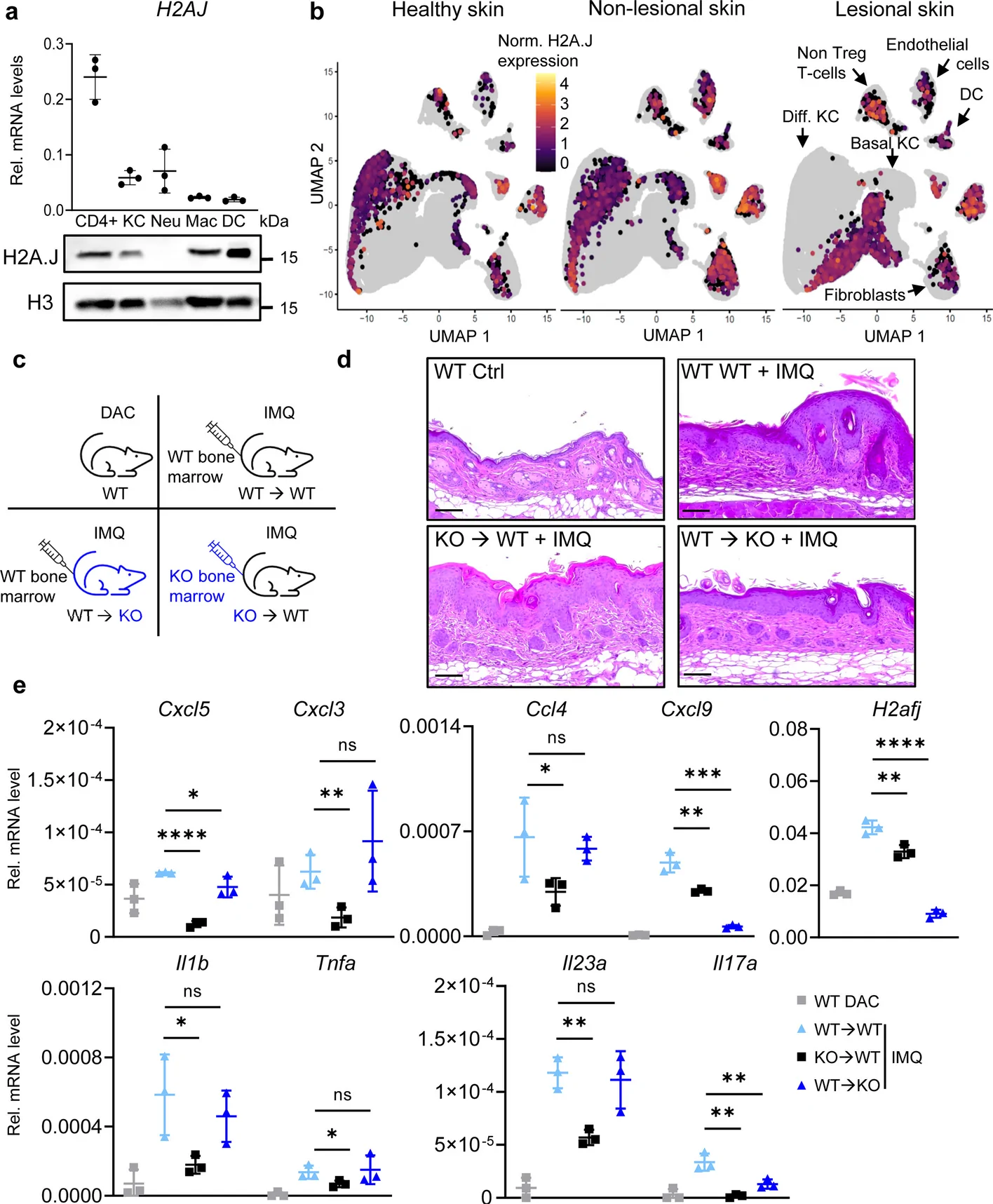
Assessment of cell-type-specific H2A.J expression. **a** mRNA and protein levels of H2A.J expression in different isolated immune cells of WT mice normalized to *Actb* or β-Actin (*n* = 3 technical replicates for gene expression analysis, mean ± standard deviation), respectively. CD4^+^ T-cells and Ly6G.^+^ neutrophils were isolated from the spleen using magnetic beads. Mouse keratinocytes were isolated from the tails of mice, and macrophages and DCs were generated from bone marrow cells. **b** UMAP plots of single-cell sequencing data derived from human healthy skin and non-lesional and lesional skin. The data are derived from a previously published dataset, which was reclustered and analyzed for keratinocyte-, T-cell-, DC-, endothelial cell-, melanocyte-, fibroblast-, smooth muscle cell, and pericyte-specific gene expression of *H2AJ* (GSE173706) [26]. Annotation of the different cell types and *H2AJ* mRNA expression levels across all patients can be found in Fig. S4. **c** Treatment scheme and the resulting groups of bone marrow chimeras. The control groups are non-chimeric mice treated with DAC cream and WT chimeric mice treated with IMQ, both with WT bone marrow. Further groups are KO chimeric mice with WT bone marrow and WT chimeric mice with KO bone marrow, both treated with IMQ. **d** H&E staining of the skin from the mice in **c** treated with DAC cream or IMQ at day 6. Scale: 100 μm. **e** RT-qPCR of the skin of the groups from **c** treated with DAC or IMQ at day 6. Relative mRNA levels were normalized to the reference gene *Rpl37a.* Shown is the mean of 3 biological replicates ± SEM. Shown is the mean ± SEM. Significance was calculated using a 2-tailed Student’s t-test (*p < 0.05, **p < 0.01, ***p < 0.001, ****p < 0.0001, ns = not significant)

In light of the findings from the mouse model, we aimed to determine which cell types express H2A.J, particularly in psoriasis. Gene expression analysis and immunoblotting showed H2A.J expression in untreated keratinocytes, T-cells, macrophages, and DC. Neutrophils lacked H2A.J protein levels, though this might also be a loading artifact (Fig. 6a). Notably, H2A.J protein expression was also detectable in the epidermis and dermis of IMQ-treated WT mice, especially in CD45-positive, dermal immune cells (Fig. S4a). Furthermore, elevated H2AJ mRNA levels were detectable in a variety of cell types in human psoriatic lesions compared to healthy skin, including basal and differentiated keratinocytes, DCs, and T-cells (Fig. 6b, Fig. S4b-d).

Given our finding that both keratinocytes and immune cells express H2A.J in psoriasis, we wanted to assess whether H2A.J expression is predominantly needed in tissue-resident cells or immune cells in order to promote IMQ-mediated psoriasis. For this purpose, we generated bone marrow chimeras harboring the deletion of H2A.J either in immune cells by engrafting H2A.J KO bone marrow into irradiated wild-type mice, or in tissue-resident, irradiation-insensitive cells by engrafting wild-type bone marrow into irradiated H2A.J KO mice (Fig. 6c). As a control, irradiated wild-type mice were reconstituted with wild-type bone marrow. Successful engraftment was controlled by immunoblot detection of H2A.J in the bone marrow or skin of the engrafted mice at the endpoint of the experiment (Fig. S5a). After 4 weeks of engraftment, mice were subsequently treated with IMQ for 6 consecutive days. Notably, ear thickness measurements and histological assessment of the treated skin at the endpoint on day 6 revealed reduced psoriasis-like skin inflammation, hyperkeratosis, and immune cell infiltration in both bone marrow chimera groups with H2A.J deletion (Fig. 6d, Fig. S5b and c). Further gene expression analysis revealed a significant reduction of some cytokines, such as *Cxcl9* and *Il17a*, in the IMQ-treated skin of both bone chimera mice with H2A.J deletion. However, most chemokines and cytokines were significantly downregulated only in mice lacking H2A.J in the bone marrow (Fig. 6e). This implies that H2A.J is needed in both tissue-resident and immune cells to promote psoriasis-associated inflammation. However, because T-cell-derived IL-17A is a key driver of psoriasis, a potential role for H2A.J in immune cells, especially T-cells, seems to be more pronounced, as most of the analyzed cytokines and chemokines were only significantly downregulated in mice harboring H2A.J KO in immune cells.

Notably, subsequent FACS analysis even revealed a significantly stronger infiltration of monocytes into the skin of mice harboring H2A.J KO immune cells, whereas H2A.J KO mice harboring wildtype immune cells displayed fewer neutrophils and significantly fewer IL-17A-expressing γδ T-cells upon IMQ treatment (Fig. S5d). Furthermore, whereas systemic levels of monocytes and neutrophils were not affected in the H2A.J KO bone marrow chimera mice, IL-17A-producing CD3^+^ T-cells were significantly reduced by the deletion of H2A.J in the bone marrow (Fig. S5e). Together, these data validate our hypothesis that H2A.J expression is needed in several cell types to fully promote psoriasis. This includes keratinocytes, for example. Given the immune cell intrinsic effect of H2A.J on IL-17A expression and the reduced presence of IL-17A-expressing T-cells only in mice lacking H2A.J in the immune cell compartment, we suggest that it might also intrinsically regulate dendritic cell and/or T_H_17 effector gene expression and function.

### Keratinocyte-derived H2A.J regulates the expression of a subset of IL-17-responsive target genes

**Fig. 7:**
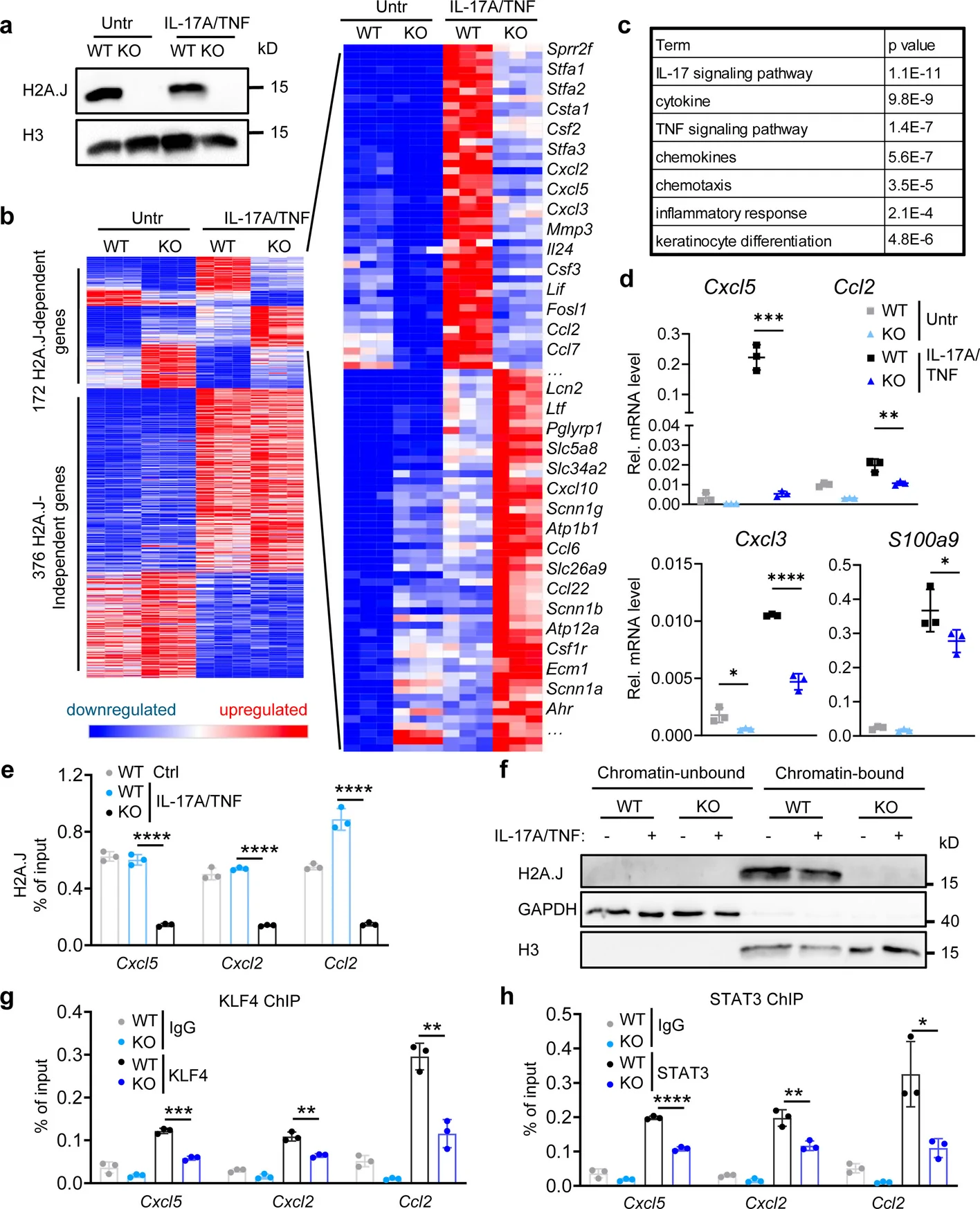
Keratinocyte-derived H2A.J regulates the expression of a subset of IL-17A-responsive target genes.** a** H2A.J protein levels in mouse keratinocytes, either left untreated or treated with 200 ng/mL IL-17A and 10 ng/mL TNF for 24 h. H3 as a loading control. **b** Transcriptome analysis of mouse keratinocytes, treated as in (**a**). The heatmap displays all genes that were either u*p-* (red) or downregulated (blue) by IL-17A and TNF treatment. (minimum fold change 2, *p-*value < 0.05 (classical t-test)). The zoom-in of the heatmap depicts all H2A.J-dependent genes. Data derived from 3 biological replicates per group. **c** Selected results from a gene set enrichment analysis of all IL-17A/TNF-induced and H2A.J-dependent target genes using DAVID bioinformatics. **d** Gene expression analysis of selected H2A.J target genes in untreated and IL-17A/TNF-treated keratinocytes. Treatment as in (**a**). Relative mRNA levels were normalized to *Actb*. Shown is the mean of 3 biological replicates ± (SD) standard deviation. **e** Chromatin immunoprecipitation (ChIP) assays of H2A.J in mouse keratinocytes of WT and H2A.J KO mice (untreated WT and treated as in (**a**) WT and KO), derived from 3 biological replicates. Shown is the mean ± SD. **f** Chromatin fractionation of WT and H2A.J knockout mouse keratinocytes treated as in (**a**). GAPDH and histone H3 served as internal controls for the chromatin-unbound and chromatin-bound fractions, respectively. **g** + **h** ChIP assays of IgG (as control) and KLF4 (**g**) and STAT3 (**h**) in mouse keratinocytes of WT and H2A.J KO mice, all treated as in (**a**), derived from 3 biological replicates. Shown is the mean ± SD. Significance was calculated using a 2-tailed Student’s t-test (*p < 0.05, **p < 0.01, ***p < 0.001, ****p < 0.0001)

To this point, we learned that key cells in psoriatic pathogenesis [27, 28], especially keratinocytes, strongly express H2A.J. Furthermore, psoriasis-associated gene expression is partially driven by H2A.J expression in tissue-resident cells, likely keratinocytes. To learn more about the gene expression effects of H2A.J in keratinocytes, we therefore isolated keratinocytes from wild-type and H2A.J KO mice and analyzed global gene expression in untreated and 24-h-treated IL-17A and TNF-stimulated cells. This stimulation represents a psoriatic milieu in cell culture settings [29] (Fig. 7a). In untreated keratinocytes, deletion of H2A.J only deregulated the expression of 88 genes, of which 33 became significantly downregulated. However, these downregulated genes mostly comprised IL-17-responsive chemokines involved in psoriasis signaling (Fig. S6a and S6b). Upon stimulation with IL-17A and TNF, 548 genes became significantly induced or repressed in WT murine keratinocytes (minimal fold change of 2, *p* ≤ 0.05), from which 172 genes became deregulated upon H2A.J deletion (Fig. 7b). Further gene set enrichment analysis revealed a strong downregulation, particularly of genes previously linked to psoriasis-associated skin inflammation, including *Cxcl5*, *Cxcl2*, and *S100a9* [30, 31], which are again key IL-17A-responsive genes (Fig. 7c and d). Notably, similar genes were affected in H2A.J knockout keratinocytes that were treated only with IL-17A (Fig. S6c). At the same time, the expression of anti-inflammatory or T_H_2-associated genes, such as *Ahr*, *Il18*, *Il33, or Ccl22,* was elevated in IL-17A/TNF-stimulated and H2A.J-deleted keratinocytes (Fig. S6d and S6e). Importantly, expression of the IL17 receptor (encoded by *Il17ra and Il17rc*) remained unperturbed in stimulated H2A.J KO keratinocytes, thereby ruling out the possibility that H2A.J regulates IL-17 receptor expression itself (Fig. S6f and S6g). Instead, H2A.J localized directly to the promoters of its target genes (Fig. 7e and f). Furthermore, as a consequence of H2A.J deletion, we detected less H3K4 trimethylation (H3K4me3) at the promoter regions of H2A.J target genes (Fig. S6h), but also less binding of KLF4 and STAT3 (Fig. 7g and h), two key transcription factors known to be related to psoriasis-associated gene expression [30, 32].

Thus, H2A.J is a previously unrecognized key epigenetic regulator of IL-17A-dependent gene expression in keratinocytes, directly binding to the promoter regions of chemokines and cytokines and subsequently modifying their overall accessibility. At the same time, keratinocyte-derived H2A.J represses the expression of anti-inflammatory molecules and those that promote T_H_2-like inflammation.

### H2A.J intrinsically regulates the function of T_H_17 cells

Apart from a strong H2A.J expression in keratinocytes, we also detected increased expression of H2A.J in DCs, macrophages, and T cells (Fig. 6). Furthermore, due to the observed effects of H2A.J deletion in the bone marrow of IMQ-treated mice, we hypothesized that H2A.J might intrinsically regulate the function and activity of dendritic cells, macrophages, or T_H_17 cells. Therefore, we decided to better characterize the molecular effects of H2A.J deletion on DC, macrophage, and T helper cell function.

For this purpose, we differentiated DCs or macrophages from bone marrow precursors and analyzed the effects of H2A.J deletion on their function upon stimulation with resiquimod, a TLR7/8 agonist commonly used in cell culture experiments for cell activation (Fig. S7). Interestingly, H2A.J did not significantly alter the expression levels of DC-derived T_H_17-polarizing cytokines, including *Il6*, *Tnfa, Il12a,* and *Il23a*, whereas deletion of H2A.J in bone marrow-derived macrophages slightly deregulated the expression of these key effector molecules (Fig. S7a). Furthermore, we observed a mild down- or upregulation of the co-stimulatory surface receptors CD86 and MHC class II, respectively in untreated, but not activated DCs and macrophages (Fig. S6b). This might imply that H2A.J-deleted dendritic cells or macrophages might affect T-cell proliferation. However, subsequent cocultivation experiments revealed no or just mild effects on T-cell proliferation in the presence of H2A.J KO DCs or macrophages, compared to wild-type cells. (Fig. S6c). Thus, H2A.J is not likely to regulate T_H_17 differentiation or activation via DCs or macrophages.

Notably, we also detected strong expression of H2A.J in untreated or PMA/Ionomycin-activated CD4^+^ T-cells, as well as in different T helper cell subsets (Fig. S8a and b), together with significantly lower T-cell-derived IL-17A expression in IMQ-treated H2A.J KO mice. This raises the possibility that H2A.J affects T_H_17 cell differentiation and function not only via DC and macrophages, but also through an intrinsic regulation of T_H_17 cell-specific gene expression.

**Fig. 8:**
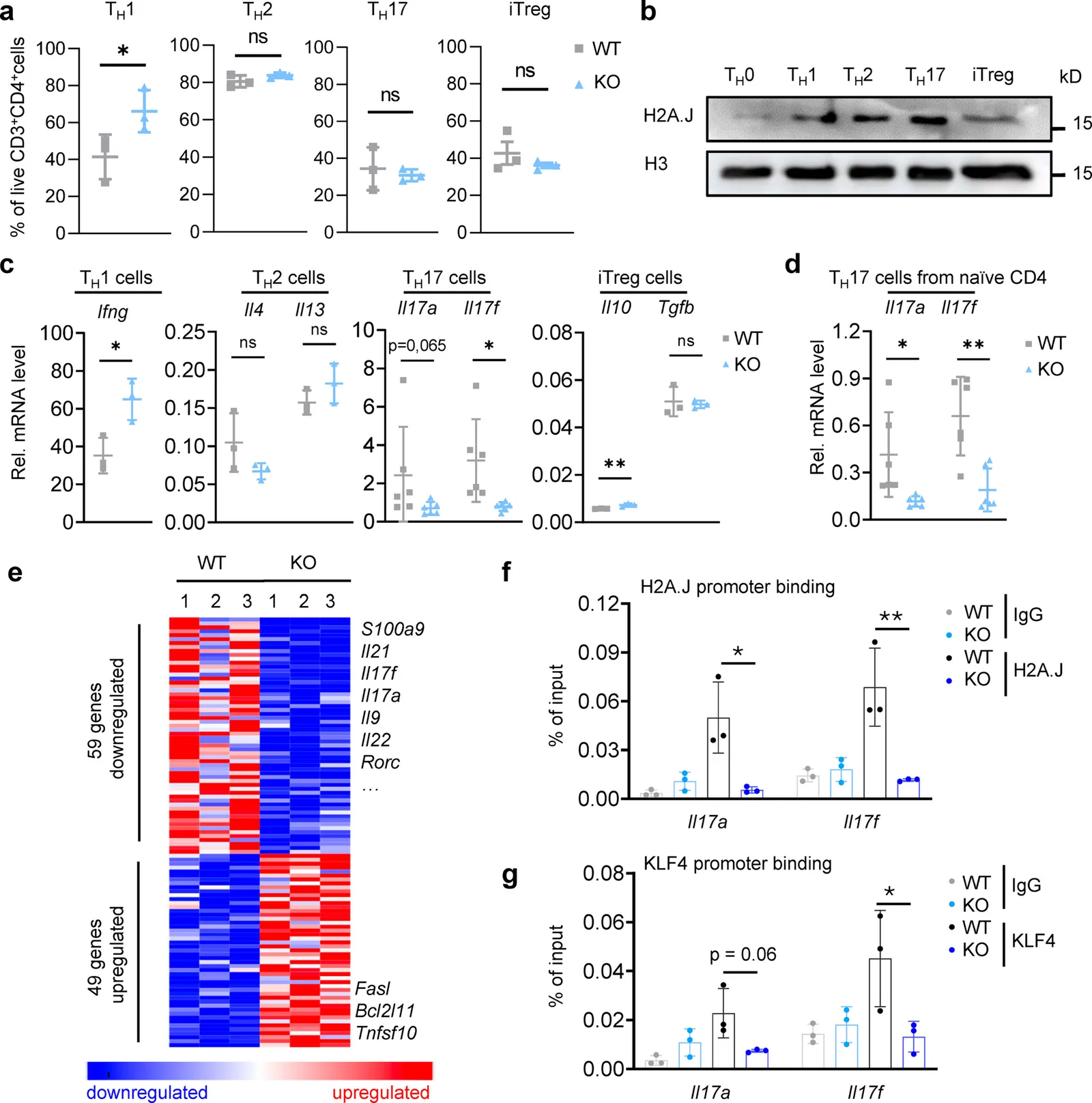
H2A.J regulates the expression of a subset of T_H_17 effector molecules. **a** Flow cytometry analysis for T helper cell differentiation using WT and H2A.J KO CD4^+^ cells. Shown is the percentage of positive cells of 3 biological replicates ± SD, after pre-gating on viable CD3^+^CD4^+^ cells. Gating markers are shown in Fig. S8b. **b** Protein levels of H2A.J in different T helper cell subsets. H3 served as a loading control. **c** Gene expression analysis of key effector molecules in in vitro differentiated T helper cell subsets of WT and H2A.J KO mice. Relative mRNA levels were normalized to *Actb*, derived from 3 biological replicates per group. Shown is the mean ± SD. **d** *Il17a* and *Il17f* mRNA expression in T_H_17 cells derived from naïve CD4.^+^ T-cells. Relative mRNA levels were normalized to *Actb*, derived from 3 biological replicates. Shown is the mean ± SD. **e** Heat map of RNA sequencing showing relevant genes upregulated (red) or downregulated (blue) after KO of H2A.J in TH17 cells differentiated from naïve CD4 + cells, from 3 biological replicates of WT or KO. H2A.J-regulated genes were defined based on a minimum fold change of 2 and a *p-*value ≤ 0.05 (Baggerley test). **f** + **g** ChIP assays of IgG (as control) and H2A.J (**f**) and KLF4 (**g**) in T_H_17 cells of WT and H2A.J KO mice, derived from 3 biological replicates. Shown is the mean ± SD. Significance was calculated using a 2-tailed Student’s t-test (*p < 0.05, **p < 0.01, ns = not significant)

To test this hypothesis, we isolated CD4^+^ T cells from the spleen of wild-type and H2A.J KO mice (Fig. 8c) and differentiated these cells into T_H_0, T_H_1, T_H_2, T_H_17, and iTreg cells (Fig. 8d). Overall, differentiation of CD4^+^ T cells into distinct T helper cell subsets was not impaired by H2A.J deletion, although we noticed a tendency towards increased generation of IFNγ-producing T_H_1 cells (Fig. 8a). Interestingly, T_H_17 cells exhibited the highest H2A.J protein levels among the T_H_ subsets (Fig. 8b). Furthermore, H2A.J deletion severely diminished the levels of the core effector cytokines *Il17a* and *Il17f* in T_H_17 cells, whereas other important effector molecules of the other T helper subsets remained unperturbed, except for elevated *Ifng* expression in T_H_1 cells (Fig. 8c). Similar results were observed with T_H_17 cells derived from the differentiation of naïve CD4^+^ T cells (Fig. 8d), and transcriptome analysis of control and H2A.J-deleted T_H_17 cells confirmed an H2A.J-dependent regulation of a subset of key effector molecules, thereby affecting their overall functionality (Fig. 8e, Fig. S8f). Finally, consistent with the results we observed in keratinocytes, H2A.J localized directly to the promoter regions of *Il17a* and *Il17f* (Fig. 8f), subsequently promoting the binding of KLF4 (Fig. 8g) and, partially, cJun and STAT3 (Fig. S8g and S8h). Thus, H2A.J is a specific epigenetic regulator in T_H_17 cells that promotes Il17a and Il17f expression, potentially by promoting KLF4 binding, a key transcription factor in T cells that induces *Il17a* and *Il17f* expression [31]. At the same time, H2A.J concomitantly seems to repress *Ifng* expression in T_H_1 cells.

In summary, we identified H2A.J as a novel epigenetic factor that is overexpressed in psoriasis. H2A.J promotes psoriatic inflammation by directly localizing to promoter regions of its target genes, thereby inducing the expression of a subset of pro-inflammatory cytokines and chemokines, predominantly in keratinocytes and TH17 cells, but potentially also in other cell types. Consequently, H2A.J deletion especially impacts T_H_17 and keratinocyte activation and function in psoriasis.

## Discussion

Histone variants constitute a recently identified epigenetic regulatory mechanism of gene expression that affects multiple biological processes, such as DNA damage repair, cell fate transition, and chromosomal integrity [9]. While dysregulated histone variant expression has already been identified as an emerging mechanism driving cell transformation, tumorigenesis, and therapy resistance in cancer, its impact on the immune system remains poorly understood [33].

Here, we analyzed the role of a poorly studied H2A histone variant, called H2A.J, in the common inflammatory skin disease psoriasis. We revealed that increased expression of H2A.J in keratinocytes and T_H_17 cells promotes the expression of psoriasis-associated cytokines and chemokines, including chemotactic proteins involved in neutrophil recruitment [34]. Importantly, we could recapitulate these findings in two independent psoriasis mouse models, using either IMQ or intradermal injections of IL-17A and IL-36γ. Additionally, similar effects could be recapitulated in IL-17A- and TNF-stimulated mouse keratinocytes, in which deletion of H2A.J suppressed the induction of a subset of IL-17A-dependent chemokines and cytokines. This effect on IL-17A-mediated gene expression may also explain the observed inhibition in hyperkeratosis and acanthosis in IMQ- or cytokine-treated H2A.J KO mice, as IL-17A is one of the central cytokines in the skin that inhibits keratinocyte differentiation [35]. Furthermore, the diminished recruitment and activation of IL-17A-expressing T_H_17 cells in the skin of IMQ-treated H2A.J KO mice could explain the observed inhibition of IMQ treatment-induced acanthosis and hyperkeratosis, as IL-17A is known to promote abnormal proliferation of keratinocytes [36].

How H2A.J regulates these gene sets comprising cytokines and chemokines remains elusive, though. It has previously been shown that H2A.J directly localizes to certain gene promoters and enhancers, although the presence of H2A.J did not necessarily imply H2A.J-dependent gene expression regulation [11, 37]. Furthermore, structural studies and molecular dynamics simulations suggest that there are only subtle differences between nucleosomes containing unmodified H2A.J compared to canonical H2A [38, 39]. However, interaction of specific transcription factors with H2A.J-specific residues, potentially after phosphorylation of its C-terminal tail, could ultimately determine whether a gene is being transcribed [40]. Consistently, we could also detect direct binding of H2A.J to its target gene promoters in keratinocytes but also in T_H_17 cells. Subsequently, loss of H2A.J partially perturbed the binding of key transcription factors, such as KLF4, cJun, and STAT3, to the promoter regions of H2A.J target genes. Whether the effect of H2A.J is related to any specific transcription factor remains to be investigated in future experiments, though. Notably, previous reports already showed that the net effect of H2A.J is highly dependent on the initial stimulus and can even result in opposing effects [11, 12, 14, 17]. Thus, we propose that the effect of H2A.J on gene expression probably depends on the initial stimulus and thus on the presence and activation of specific transcription factors. Furthermore, it needs to be investigated whether H2A.J is also post-translationally modified in activated keratinocytes and T_H_17 cells, and whether this further modifies the recruitment of specific transcription factors to control gene expression in psoriasis.

As previously described, H2A.J expression could be detected in the epidermis [13], but we also found H2A.J-positive immune cells within the dermis of psoriatic skin. Further analysis confirmed strong expression of H2A.J in several immune cell types, including macrophages, DCs, and T cells. Using bone marrow chimeras that either harbored a deletion of H2A.J in tissue-resident cells or in the bone marrow, we revealed an important role for H2A.J in both compartments in psoriasis. As deletion of H2A.J in tissue-resident cells and in the bone marrow partially diminished psoriasis-associated skin inflammation. Notably, the phenotype of IMQ-treated bone marrow chimeras harboring the H2A.J KO in the immune cells was more pronounced than in mice harboring a tissue-resident H2A.J deletion. This implies a key role for H2A.J in immune cell function. In subsequent experiments, we could not reveal a significant impact of H2A.J on DC or macrophage activity or function, at least when we analyzed its role in resiquimod-stimulated BMDC or BMDM in vitro. Instead, we observed a significant impact of H2A.J deletion on T helper cell function, as H2A.J directly bound to the promoter regions of *Il17a* and *Il17f,* subsequently promoting their expression in T_H_17 cells. At the same time, *Ifng* expression in T_H_1 cells was surprisingly repressed by H2A.J. Together, these data imply that T-cell-derived H2A.J specifically controls the gene expression of effector molecules in T_H_1 and T_H_17 cells, subsequently contributing to the onset of psoriasis at least through a strong promotion of IL-17A and IL-17F expression in T_H_17 cells. Here, future experiments using T-cell-specific H2A.J knockout are needed to validate this hypothesis, possibly not only in the setting of psoriasis, but also in other T_H_17-driven diseases.

Our data validate previous findings from studies investigating other histone H2A variants, which were found to promote and repress distinct cytokine response programs, at least in tumor cells. For example, macroH2A has recently been identified as an inhibitor of IFN-STAT signaling in tumor cells, whereas TNF-NF-κB signaling is promoted by macroH2A [41]. Similarly, H2A.Z has previously been identified as an inhibitor of the IFN response in HeLa cells [42]. We identified H2A.J as both an inhibitor of *Ifng* expression and a stimulator of *Il17a* and *Il17f* expression. It therefore follows that H2A.J, but possibly also other histone variants, regulate specific cytokine-dependent signaling programs and consequently influence immune cell plasticity and function [11, 43, 44]. As it is already established that different histone variants are expressed in multiple immune cell subsets and that their expression changes during immune system aging [26], histone variants might have a significant impact on immune cell function and activity. Furthermore, as demonstrated in this study of H2A.J and its role in psoriasis, dysregulated histone variant expression could potentially promote the polarization of tissues into T_H_1-, T_H_2-, or T_H_17-like states, subsequently contributing to the onset of multiple autoinflammatory diseases. It will therefore be very interesting to investigate H2A.J, but also other histone variants and their impact on T_H_1, T_H_2, or T_H_17-related autoimmune and autoinflammatory diseases, such as colitis, atopic dermatitis, allergic asthma, or multiple sclerosis.

In summary, we identified the histone variant H2A.J as a new epigenetic factor that promotes IL-17-associated inflammation in psoriasis by regulating cytokine and chemokine expression in keratinocytes and T_H_17 cells. Of course, this study is limited by the use of only global H2A.J KO mice, which does not allow us to dissect the cell-type-specific role of H2A.J in psoriasis. Furthermore, it remains an open question whether elevated H2A.J levels precede the onset of psoriasis, or whether skin inflammation promotes elevated H2A.J levels. Therefore, future research should assess the cell-type-specific role of H2A.J in psoriasis, and further investigate H2A.J in human psoriasis pathology. Moreover, it has to be clarified whether high H2A.J levels in healthy skin predispose to psoriasis and whether they also contribute to disease persistence and imprinting at lesional sites.

## Material and methods

### Mice

Sex as a biological variable: For all experiments, the two lines were sex- and age-matched (approximately 10–14 weeks old). Both sexes were used, and no difference between them could be detected. All experiments involving animals were approved by the local animal ethics committee (Landesuntersuchungsamt Rheinland-Pfalz; G22-01–081). Imiquimod-induced psoriasis-like inflammation was conducted as previously described [45]. In brief, Aldara® cream (5% IMQ, 3 M Pharmaceuticals) was applied to both ears and the back skin (1 × 1 cm) on 6 consecutive days, and a vehicle basic cream (DAC) was applied to the global *H2afj* KO and wildtype mice. On day 6, the mice were sacrificed and analyzed. The bone marrow-chimeric mice were irradiated with a dose of 10 Gy for 20 min (X-ray machine: Precision X-Rad 320 Spellman MXR321) 4 weeks before the start of treatment with Aldara® cream. After irradiation, the mice received a bone marrow transplant. For this purpose, bone marrow was harvested from the femurs of donor mice, and 0.5 × 10⁷ cells in PBS were injected into the irradiated recipient mice via the tail vein. Starting 2 days before radiation treatment and continuing through the 10th day after radiation, the recipient mice were given 0.1% Borgal (0.833 g of sulfadoxine and 0.167 g of trimethoprim per liter) in their drinking water to prevent spontaneous infections in these immunocompromised mice. As a second psoriasis-like inflammation model, 1 µg of recombinant murine IL-17A (Immunotools; Cat. 12340176) and 1 µg of IL-36γ (BioLegend; Cat. 552806) dissolved in 10 µL of PBS were injected intradermally into both ears of the animals. The injections were administered on four consecutive days. Afterward, the mice were euthanized and analyzed the following day. As a control, 10 µL of PBS was injected into control mice, and mice were euthanized in parallel.

The *H2afj* KO mice have a TALEN-mediated deletion of 7 nucleotides at the start of the *H2afj* gene that creates a frameshift mutation after the fourth amino acid of H2A.J and introduces a premature stop codon [17]. The mutant mice were generated at Cyagen. The *H2afj* KO mice (H2A.J KO) were obtained from Carl Mann (University of Paris-Saclay, France), and frozen mutant sperm were deposited at the European Mutant Mouse Archive (EMMA ID 13660). C57BL/6NCrl mice were derived from backcrosses of C57BL/6N-H2A.J KO and C57BL/6NCrl and were subsequently used as control mice to avoid inbred artifacts.

### Statistics

Samples generated from cell culture are presented as mean ± standard deviation (SD), whereas samples from mouse experiments are presented as mean ± standard error of the mean (SEM, *n* = number of animals). Unless otherwise stated, significance was calculated using a 2-tailed Student's t-test and is represented by asterisks (*p < 0.05, **p < 0.01, ***p < 0.001, and ****p < 0.0001). All data blots were generated using the software GraphPad Prism.

All additional materials and methods are explained in the Supplementary Methods.

## Supplementary Information

Supplementary Material 1.

## Data Availability

The raw and processed data of bulk RNA sequencing can be found under GEO accession numbers: GSE307885 (T_H_17 data set) and GSE307884 (mKC data set). Single-cell sequencing analysis data and bulk RNA sequencing data of human psoriasis samples were downloaded from Gene Expression Omnibus (Accession numbers: GSE173706 and GSE13355).
