# Supplementary material for: H2A.J is an epigenetic driver of IL-17-associated inflammation in TH17 cells and keratinocytes
Source: Mol Biomed. 2026 Oct 8;7:199. doi: 10.1186/s43556-026-00611-4 (PMC13650110; doi:10.1186/s43556-026-00611-4)
Supplement: Supplementary file 1 [file 43556_2026_611_MOESM1_ESM.docx]

**H2A.J is an epigenetic driver of IL-17-associated inflammation in T_H_17 cells and keratinocytes**

Tanja Kübelbeck^1^, Anna Stastny^1^, Niklas Beumer^2,3^, Antonia Kolb^1^, Tanja Knopp^4,5^ Berenice Fischer^1^, Dennis Fritsche^1^, Ana-Marija Kulis-Mandic^1^, Matthias Klein^2,3^, Mariia Antipova^6^, Bettina Mros^5^, Michael Delacher^2,3^, Ari Waisman^3,6^, Carl Mann^7^, Claudia E. Rübe^8^, Susanne Karbach^4,5,9^, Björn E. Clausen^3,5^, Carsten Deppermann^3,5^, Miriam Wittmann^1,3^, Daniela Kramer^1,3,*^

^1^ Department of Dermatology; University Medical Center of the Johannes Gutenberg-University Mainz; Germany

^2^ Institute of Immunology; University Medical Center of the Johannes Gutenberg-University Mainz; Germany

^3^ Research Center for Immunotherapy; University Medical Center of the Johannes Gutenberg-University Mainz; Germany

^4^Center for Cardiology-Cardiology I, University Medical Center of the Johannes Gutenberg University Mainz, Germany

^5^ Center for Thrombosis and Hemostasis; University Medical Center of the Johannes Gutenberg University Mainz; Germany

^6^ Institute for Molecular Medicine; University Medical Center of the Johannes Gutenberg-University Mainz; Germany

^7^ Université Paris-Saclay, CEA, CNRS; Institute for Integrative Biology of the Cell (I2BC), 91198 Gif-sur-Yvette; France

^8^ Department of Radiation Oncology; Saarland University Medical Center; Homburg/Saar; Germany

^9^ German Center for Cardiovascular Research, Partner Site Rhine-Main, Mainz, Germany

^*^Corresponding author:

Prof. Dr. Daniela Kramer, University Medical Center of the Johannes-Gutenberg University Mainz, Department of Dermatology, Langenbeckstr. 1, 55131 Mainz, Germany, Email: [kramerda@uni-mainz.de](mailto:kramerda@uni-mainz.de), Tel.: +496131/17-5731.

**Supplementary Information**

**Figure S1.** Additional data on untreated and IMQ-treated H2A.J KO mice.

**Figure S2.** Additional data on IL-17A- and IL-36γ-treated mice.

**Figure S3.** Neutrophil and monocyte numbers in the bone marrow of IMQ-treated WT and H2A.J KO mice.

**Figure S4.** Extended data on single-cell data and H2A.J staining in IMQ-treated mouse skin.

**Figure S5.** Additional data on IMQ-treated bone marrow chimeras.

**Figure S6.** More data on the effects of H2A.J in keratinocytes.

**Figure S7.** Effect of H2A.J KO in DC and macrophages

**Figure S8.** Additional data on H2A.J deletion in T-cells.

**Supplementary Methods.**

**Table S1.** ChIP and CUT&RUN expression primer.

**Table S2.** Gene expression primer.

**Figure S1.** **Additional data on untreated and IMQ-treated H2A.J KO mice.** (**a**) Pictures of 11-week-old WT (C57BL/6N) and H2A.J KO mice. (**b**) Body weight of female and male WT and H2A.J KO mice. Shown is the mean of 6 (female) or 8 (male) biological replicates ± SEM per group. (**c**) Individual data points of the ear thickness from Fig. 2c at day 0 and 6. Shown are 6 biological replicates ± standard error (SEM). (**d**) Cumulative Psoriasis Area and Severity Index (PASI) score during the course of the experiment. A score of 0 = no signs, 1 = very mild, 2 = mild, 3 = moderate, 4 = severe, and 5 = very severe (including 0.5 for values between two scores) is assigned each day for the following criteria: ear thickness, erythema, and scaling. The cumulative PASI score is calculated by adding the three values together. Shown are 6 biological replicates ± standard error (SEM) per group. (**e**) Individual data points of the skin thickness from Fig. 2 at day 6. The figure shows several measurements of epidermal thickness based on histological images of 6 biological replicates ± standard error (SEM). (**f**) H&E staining of back skin from WT and H2A.J KO mice treated with DAC or IMQ. Scale: 100 µm. (**g**) Flow cytometry plots of immune cells infiltrating the skin or producing IL-17A from WT and H2A.J KO mice, treated with DAC or IMQ at day 6, related to Fig. 2f + g. Shown are the gating strategy and examples of each staining for all experimental groups. Significance was calculated using a 2-tailed Student’s t-test (**p* < 0.05, ****p < 0.0001, *ns* = not significant).

**Figure S2.** **Additional data on IL-17A- and IL-36γ-treated mice.** (**a**) Individual data points of the ear thickness from Fig. 4b at day 0 and 4. Shown are 6 biological replicates ± standard error (SEM) per group. (**b + c**) Immunohistochemical staining of (**b**) MPO (myeloperoxidase) and (**c**) pRB (phosphorylated retinoblastoma protein) in ear samples from WT and H2A.J KO mice upon treatment with PBS or IL-17A and IL-36γ at day 4. Scale: 100 μm

**Figure S3.** **Neutrophil and monocyte numbers in the bone marrow of IMQ-treated WT and H2A.J KO mice.** (**a**) Individual data points for the weight change in Fig.5a at days 0 and 6. Shown are 8 biological replicates ± standard error (SEM). (**b**) Flow cytometry analysis with corresponding plots of neutrophils and monocytes in the bone marrow from DAC and IMQ-treated WT and H2A.J KO at day 6. Shown is the number of positive cells from 3 biological replicates ± SEM after pre-gating on viable CD45^+^ cells. Significance was calculated using a 2-tailed Student’s t-test (**p* < 0.05, **p < 0.01, *ns* = not significant).

**Figure S4.** **Extended data of the single-cell data and H2A.J staining in IMQ-treated mouse skin.** Related to Fig. 6a + b. (**a**) Immunofluorescence staining of H2A.J in IMQ-treated WT and H2A.J KO mice at day 6. H2A.J is in red, and CD45 is in purple. Epidermis (epi) and dermis are marked. The overlay (merge) is zoomed in on the marked area. (**b**) Dot plot showing the expression of the different markers that were used to identify T cells (CD45^+^, CD3D^+^, CD3E^+^, TRAC^+^), basal keratinocytes (KRT5^+^, KRT14^+^), differentiated keratinocytes (KRT1^high^, KRT10^high^ and further differentiated into FLG^low/high^, LOR^low/high^) and dendritic cells/ Langerhans cells (CD1A^+^, CD207^+^, CD11b^+^, CD11C^+^, HLA-DRA^+^), endothelial cells (CD31^+^, VWF^+^, EMCN^+^), melanocytes (MITF^+^, PMEL^+^, MLANA^+^, TYR^+^), smooth muscle cells and pericytes (ACTA2^+^, CNN1^+^, MYH11^+^, TAGLN^+^, RGS5^+^, PFGFRB^+^), Schwann cells (MBP^+^, MPZ^+^, PLPL1^+^, S100B^+^, NGFR^+^, SCN7A^+^, L1CAM^+^, NCAM1^+^), fibroblasts (PDGFRA^+^, LUM^+^, COL1A1^+^, COL1A2^+^, COL6A2^+^) and other (not expressing any of the before mentioned markers). (**c**) UMAP plot showing the clustering of the analysis based on the markers described in (**b**). (**d**) *H2AJ* mRNA expression analysis in single-cell sequencing data of healthy skin and non-lesional and lesional skin from psoriasis patients (n = 8 healthy skin, n = 11 non-lesional skin, n = 16 lesional skin).

**Figure S5.** **Additional data on IMQ-treated bone marrow chimeras.** Related to Fig. 6c-e. (**a**) Protein levels of H2A.J in the bone marrow and the skin at the endpoint of non-chimeric mice treated with DAC cream, WT chimeric mice having WT bone marrow (WT 🡪 WT), KO chimeric mice with WT bone marrow (WT 🡪 KO), and WT chimeric mice with KO bone marrow (KO 🡪 WT); the last three were treated with IMQ. H3 served as a loading control. (**b**) *Left:* Ear thickness during the course of the experiment. Shown is the mean of 3 to 6 biological replicates ± standard error (SEM); *Left*: ear thickness measurements over time. *Right:* Individual ear thickness data points at day 0 and 6; shown are 3 to 6 biological replicates ± standard error (SEM). (**c**) H&E staining of the ears from the mice in (**a**) treated with DAC cream or IMQ at day 6. Scale: 100 μm. (**d**) Flow cytometry analysis of skin-infiltrating immune cells at day 6 upon DAC or IMQ treatment of the mice described in (**a**). *Left:* the percentage of viable, positive cells. The following stainings were applied: neutrophils: Ly6C^+^ Ly6G^+^, monocytes: Ly6G^-^, Ly6C^+^, and *right:* the percentage of viable, CD45^+^ CD3^+^, positive cells. The following staining was applied: γδ^+^ and IL-17A^+^. Depicted is the average of 3 biological replicates ± standard error (SEM). (**e**) Flow cytometry analysis of immune cells in the spleen at day 6 upon DAC or IMQ treatment of the mice described in (**a**). *Left*: percentage of viable, CD45^+^ CD3^+^ cells. The following stainings were applied: γδ^+^ and IL-17A^+^. *Right:* percentage of viable CD45^+^ cells. The following stainings were applied: neutrophils: Ly6C^+^ Ly6G^+^, monocytes: Ly6G^-^, Ly6C^+^. Depicted is the average of 3 biological replicates ± standard error (SEM). Significance was calculated using a 2-tailed Student’s t-test (**p* < 0.05, ****p* < 0.001, *ns* = not significant).

**Figure S6.** **More data on the effects of H2A.J in keratinocytes.** (**a**) Zoom-in of the heatmap depicts all H2A.J-dependent genes in untreated keratinocytes (minimal fold change of 2, p-value ≤ 0.05. (**b**) Selected results from a gene set enrichment analysis of (**a**) using EnrichR. (**c**) Selected results from a gene set enrichment analysis of IL-17A/TNF-regulated, H2A.J-upregulated genes using DAVID bioinformatics (related to Fig. 6b). (**d**) Validation of selected genes from (**c**) that are upregulated by H2A.J in IL-17A/TNF-stimulated keratinocytes. (**e**)**.** Gene expression analysis of murine keratinocytes from WT and H2A.J keratinocytes, 24 h after stimulation with 200 ng/mL IL-17A. Relative mRNA levels were normalized to *Actb*. Shown is the mean of 3 biological replicates ± SD. (**f**) Immunofluorescence staining of IL17R (in green) and DAPI (in blue) in IL-17A/TNF-treated mouse keratinocytes from WT and H2A.J KO mice. (**g**) IL-17R expression analysis of murine keratinocytes from untreated and IL-17A/TNF-treated WT and H2A.J keratinocytes. Relative mRNA levels were normalized to *Actb*. Shown is the mean of 3 biological replicates ± SD. (**h**) Chromatin immunoprecipitation (ChIP) assays of IgG (as control) and H3K4me3 in IL-17A/TNF-treated mouse keratinocytes of WT and H2A.J KO mice, derived from 3 biological replicates. Shown are the mean ± SD. Significance was calculated using a 2-tailed Student’s t-test (*p < 0.05, **p < 0.01, ***p < 0.001, ****p < 0.0001, *ns* = not significant).

**Figure S7**. **Effect of H2A.J KO in DC** **and macrophages.** (**a**) Gene expression analysis of bone marrow-derived dendritic cells (BMDC - *top*) and macrophages (BMDM - *bottom*) from WT and H2A.J KO mice, either untreated or 24 h after stimulation with 1 µg/mL resiquimod. Relative mRNA levels were normalized to the reference gene *Rpl37a*. Shown is the mean of 3 biological replicates ± SD. (**b**) Flow cytometry analysis. Cells and treatment as in (**a**). *Top:* Shown is the percentage of positive cells as the mean of 4 biological replicates ± SD, after pre-gating on viable MHCII high CD11c^+^ cells for BMDC or F4/80^+^ CD11b^+^ cells for BMDM. *Bottom:* Gating strategy. (**C**) Assessment of CD4^+^OT-II^+^ T-cell proliferation in the presence of OVA-stimulated WT and H2A.J KO BMDC (*top*) or BMDM (*bottom*). DC-mediated T-cell proliferation was assessed by flow cytometry after labeling of the T cells with CTV. OTII T cells were cultured with WT and H2A.J KO BMDCs, preincubated with 1 µg/mL resiquimod and 100 µg/mL Ovalbumin (OVA). Shown is the percentage of positive cells in 3 biological replicates ± SD, after pre-gating on viable CD4^+^ cells. *Left:* gating strategy. *Right:* Evaluation of the percentage of T cells that proliferated between 0 - 4 cycles.

**Figure S8. Additional data on H2A.J deletion in T cells.** (**a**) Immunoblotting of H2A.J in untreated or P/I-stimulated CD4^+^ T cells. (PMA 50 ng/mL and Ionomycin 500 ng/mL). H3 served as a loading control. (**b**) *H2afj* mRNA levels in different T helper cell subsets. Treatments as described for Fig. 8a. mRNA levels were normalized to *Actb*. Shown is the mean of 3 biological replicates ± SEM. (**c**) Flow cytometry plots controlling the purity of viable, CD3^+^ CD4^+^ T cells isolated from the spleen using magnetic beads. (**d**) Flow cytometry analysis assessing the effectiveness of in vitro differentiation of CD4^+^ T cells into T helper cell subsets. After pre-gating on viable CD3+ cells, we applied the following markers: T_H_1 (IFNγ+), iTreg (FoxP3+), T_H_2 (GATA3+), and T_H_17 (RORγt+). (**e**) Examples of the flow cytometry analysis of the different T_H_ subsets after pre-gating on viable CD4^+^ cells, which were generated from isolated WT and H2A.J KO CD4^+^ T cells. (**f**) Gene set enrichment analysis of H2A.J target genes in T_H_17 cells from Fig. 8e. Analysis was done with EnrichR. (**g+h**) ChIP assays of IgG (as control) and cJun (**g**) and STAT3 (**h**) in T_H_17 cells of WT and H2A.J KO mice, derived from 3 biological replicates. Shown is the mean ± SD. Significance was calculated using a 2-tailed Student’s t-test (*ns* = not significant).

**Supplementary Methods**

**Cell culture and treatments**

Isolated primary murine keratinocytes were cultured in Keratinocyte SFM (1x) (Thermo Fisher, Cat. 17005042); T-cells from OT II mice and bone marrow-derived macrophages or DCs were cultivated in IMDM medium supplemented with 10% FCS, 100 U/ml P/S, and 50 µM β-mercaptoethanol. The keratinocyte SFM (1x) medium was supplemented with antibiotics and the supplied supplements containing human recombinant epidermal growth factor (rEGF) and bovine pituitary extract (BPE). Isolated primary T-cells and bone marrow-derived macrophages were cultivated in RPMI-1640 medium (Sigma-Aldrich, Cat. R8758-500ML), supplemented with 10% FCS, antibiotics, and for T-cells, with 50 µM ꞵ-mercaptoethanol and 1% non-essential amino acids. All cells were grown at 5% CO_2_ and 37°C in a humidified atmosphere. Primary murine keratinocytes were stimulated with 200 µg/mL recombinant murine IL-17A (Immunotools, Cat. 51872048) alone or together with 10 µg/mL murine TNF (Immunotools, Cat. 51732963). Isolated T-cells were differentiated into T helper cell subsets by various murine cytokines. All subsets were stimulated with 4 µg/mL plate-coated α-CD3 (BioLegend, Cat. 100340) and 2 µg/mL α-CD28 (BioLegend; Cat. 102116). Other cytokines that were used for differentiation of T helper subsets were the following: T_H_0: 1 µg/mL α-IFNγ (Immunotools, Cat. 22853631), 1 µg/mL α-IL-4 (BioLegend, Cat. 504102), 5 ng/mL IL-2 (Immunotools, Cat. 12340024); T_H_1: 1 µg/mL α-IL-4, 5 ng/mL IL-2, 15 ng/mL IL-12 (BioLegend, Cat. 51781527); T_H_2: 1 µg/mL α-IFNγ, 5 ng/mL IL-2, 10 ng/mL IL-4 (BioLegend, Cat. 22229021); iTregs: 1 µg/mL α-IFNγ, 1 µg/mL α-IL-4, 5 ng/mL IL-2, 15 ng/mL TGF-ꞵ (BioLegend, Cat. 763104): T_H_17: 1 µg/mL α-IFNγ, 1 µg/mL α-IL-4, 1 µg/mL α-IL-2, 2.5 ng/mL TGF-ꞵ, 50 ng/mL IL-6 (Immunotools, Cat. 12340063), 20 ng/mL IL-23 (BioLegend, Cat. 589002), 20 ng/mL IL-1β (Immunotools, Cat. 12340012). Additionally, isolated T-cells were stimulated with 50 ng/mL PMA (Sigma-Aldrich, Cat. P1585) and 500 ng/mL ionomycin (Biomol, Cat. Cay11932-1). The bone marrow-derived DCs and macrophages were stimulated with 1 µg/mL resiquimod (InvivoGen, Cat. Tlrl-r848) and partially in combination with 100 µg/mL OVA (Sigma-Aldrich, Cat. A5503).

**Isolation of primary cells**

Primary murine keratinocytes (mKC) were isolated as previously described [1]. Murine primary T-cells were isolated using the EasySep™ Mouse T-Cell Isolation Kit from Stemcell (Cat. 19851) or the EasySep™ Mouse Naïve CD4+ T-Cell Isolation Kit from Stemcell (Cat. 19765), respectively. T-cells from OTII mice were isolated using CD4 (L3T4) MicroBeads from Miltenyi Biotec (Cat. 130-117-043). The isolation of murine primary neutrophils was performed using the EasySep™ Mouse Neutrophil Enrichment Kit from Stemcell (Cat. 19762).

To isolate bone marrow-derived macrophages (BMDM) and DC (BMDC), the bone marrow from the femur and tibia was rinsed by applying PBS through a syringe with a small needle into a 70 µm strainer, which was placed into a 50 mL centrifugation tube. Erythrocytes were lysed with ACK (Ammonium Chloride (Carl Roth, Cat. P726.1) and Potassium hydrogen bicarbonate (Carl Roth, Cat. X887.1) lysis buffer for 2 min. Cells were cultured at a density of 1 x10^6^ cells/ml in RPMI 1640 medium supplemented with 10% FCS, antibiotics, 50 µM ꞵ-mercaptoethanol, and 20 ng/mL GM-CSF (produced in-house as described in [2] for BMDC generation, or 20 ng/mL murine recombinant M-CSF (Immunotools, Cat. 12343117) for BMDM culture. Culture medium was changed every 2 days. After 5 days, the BMDMs were detached from the surface by scraping, seeded in 6-well plates in medium, pre-starved in RPMI without supplements for 24 hours, and subsequently harvested on day 8. For BMDC generation, cells were cultured for 10 days and subsequently harvested after treatment for 24 hours.

**BMDC, BMDM and T-cell co-culture**

On day 9 of the BMDC and day 6 of the BMDM culture, cells were seeded in a 96-well plate in 100 μL of complete IMDM medium. Cells were pulsed with 100 µg/mL OVA (Sigma-Aldrich, Cat. A5503) and additionally stimulated with 1 µg/mL resiquimod (InvivoGen, Cat. Tlrl-r848). The next day, isolated OTII CD4^+^ T-cells expressing transgenic OVA-specific αβ-TCRs were treated with CellTrace Violet Cell Proliferation Dye (Invitrogen, Cat. C34557) according to the manufacturer’s protocol and seeded with BMDCs in a 1:10 ratio and with BMDMs in a 1:2 ratio in 200 μL of complete IMDM medium. After 4 days, cells were harvested and analyzed by flow cytometry.

**Histology**

Sex as a biological variable: Both sexes were used, and no difference between them could be detected. All experiments involving patient material were approved by the local ethics committee of Mainz (Ethikkommission der Landesärztekammer Rheinland-Pfalz, No. 2019-14135). 5 µm paraffin sections were prepared from samples that were previously fixed with 4% formaldehyde overnight. Phospho-RB (Cell Signaling, Cat. 8516) and H&E staining were performed as previously described [3]. For MPO staining, the antibody (R&D, Cat. AF3667) was incubated overnight at 4°C, at a dilution of 1:200 in 5% normal horse serum. After washing, sections were incubated with the secondary antibody (Thermo Fisher, A15999, dilution 1:500) for one hour at room temperature, washed, and finally stained with Vector NovaRED (Vector Laboratories, Cat. SK-4800). Counterstaining was performed with hematoxylin. For H2A.J staining (MyBiosource, Cat. MBS3217357), antigen retrieval was performed for 40 min in 1 mM EDTA (pH 8.0), followed by 10 min quenching in 5% H_2_O_2_ and 1 hour blocking in 5% normal goat serum. The H2A.J antibody was incubated overnight at 4°C at a dilution of 1:500. After washing, sections were incubated with the secondary antibody for one hour at RT and finally stained with Vector NovaRED. Counterstaining was performed with hematoxylin. The slides were digitized using a NanoZoomer 2.0-HT scanner (Hamamatsu Photonics, Herrsching, Germany), and the images were acquired using QuPath [4].

**Immunoblotting**

Immunoblot analysis was performed as previously described [3]. Except for the detection of H2A.J from murine samples, acid extraction was performed. For this purpose, tissue in PBS was homogenized in a gentleMACS™ M Tube (Miltenyi Biotech, Cat. 130-093-236) in the Miltenyi program Proteins_01 on a gentleMACS™ Octo Dissociator. Subsequently, 0.5% Triton X-100 and 1 x protease inhibitor cocktail (Sigma, Cat. 469312400) were added. For cells, samples were directly resuspended in PBS, supplemented with 0.5% Triton X-100 and 1x protease inhibitor cocktail. Tissue and cells were incubated for 10 min on ice. Afterwards, lysates were centrifuged (2000 rpm, for 10 min at 4 °C), and the pellets were dissolved in 0.2 M HCl. Subsequently, pellets were incubated on ice (for cell pellets: 10 min, for tissue pellets: 60 min). To stop the acid extraction, the supernatant was neutralized with 1 M NaOH. Subsequently, SDS-PAGE and immunoblotting were performed, as previously described [3]. The following antibodies were used: α-H2A.J from Active Motif (Cat. 61793), 1:1000 diluted in 5% milk/PBS, and α-H3 as a loading control from Abcam (Cat. ab1791), 1:5000 diluted in 5% milk/PBS.

**Chromatin fractionation, chromatin immunoprecipitation, and CUT&RUN**

Chromatin fractionation was performed as described by Mendez and Stillman [5]. As controls for the chromatin-unbound and chromatin-bound fractions, α-GAPDH (Cell Signaling, Cat. 2118) 1:1000 in 5% BSA and α-H3 antibodies (Abcam, Cat. ab1791) 1:5000 in milk/PBS were used, respectively. ChIP assays were performed as described before [3]. In brief, chromatin was prepared by cross-linking with 1% formaldehyde for 10 min (Thermo Fisher, Cat. 28906), followed by 20 min of sonication. The isolated chromatin was incubated with protein G-coupled Dynabeads (Thermo Fisher, Cat.10003D) and 3 µL α-H2AJ (active motif, Cat. 61793), 1 µg α-Tri-Methyl-Histone H3 (Lys4) (Diagenode, Cat. C15410003), 5 µL α-KLF4 (R&D, Cat. AF3158), 3 µL α-STAT3 (Thermo Fisher, Cat. MA1-13042) or an IgG antibody as control (Abcam, Cat. ab46540), overnight at 4 °C on a rotator.

CUT&RUN assays were performed using the CUT&RUN Assay Kit (Cell Signaling Technology, Cat. No. 86652) according to the manufacturer’s protocol. For chromatin enrichment, either an IgG isotype control antibody (Cell Signaling Technology, Cat. No. 66362) or 3 µL α-H2AJ (active motif, Cat. 61793), 3 µL α-STAT3 (Thermo Fisher, Cat. MA1-13042), 3 µL α-NF-κB p65 (Diagenode, Cat. C15310256), 5 µL α-KLF4 (Abcam, Cat. ab151733) antibody was used. After purification, ChIP and CUT&RUN samples were analyzed by qPCR. Primers for both are listed in Table S1. Maxima SYBR Green Master Mix (Thermo Fisher, Cat. K0221) was used to perform RT-qPCR; The percentage of input was calculated as described [3].

**Flow cytometry**

The preparation of flow cytometry samples from skin and bone marrow was performed as previously described [1]. For the generation of a single-cell suspension from the spleen, the whole organ was pressed through a 70 µm strainer into a 50 mL centrifuge tube. Erythrocytes were lysed with ACK (Ammonium Chloride (Carl Roth, Cat. P726.1) and Potassium hydrogen bicarbonate (Carl Roth, Cat. X887.1) lysis buffer for 2 min. For cytokine staining, the single-cell suspension (in RPMI medium supplemented with antibiotics and 10% FCS) was first stimulated with PMA/Ionomycin (see cell stimulation for concentrations) for 4 hours in combination with 1:1500 diluted GolgiStop (BD Biosciences, Cat. 554724) in a cell incubator (37°C, 5% CO2). Before staining with surface antibodies, all tissues were incubated with 1x Fc-Block (BioLegend, Cat. 101320) diluted in FACS buffer (PBS with 5% FCS and 1 mM EDTA) for 10 minutes at 4°C. Surface staining was performed at 4°C for 20 min in FACS buffer. When intracellular staining was performed, the samples were previously fixed with the FoxP3 Transcription Factor Staining Buffer Set (Invitrogen, Cat. 00-5523-00) for 1 hour at 4°C, and then washed twice in 1x permeabilization buffer. The intracellular antibodies were stained in permeabilization buffer and incubated overnight. Finally, the samples were washed in FACS buffer and resuspended in FACS buffer for analysis on the cytometer (LSRII flow cytometer, Becton Dickinson). Gates were defined by fluorescence minus one and unstained samples, and the same gates were applied for all samples. Subsequently, the data were analyzed using FlowJo.

The following antibodies were used for skin, spleen and bone marrow (all from Biolegend): α-CD3-PB (Cat. 100214), α-CD4-PerCP (Cat. 100539), α-Ly6G-PE (Cat. 127608), α-Ly6C-APC (Cat. 128016), α-F4/80-PE (Cat. 123110), α-F4/80-FITC (Cat. 123108), α-CD11c-APC (Cat. 117310), α-CD11c-PE (Cat. 117308), α-γδ-APC (Cat. 118116), α-IL-17A-PE (Cat. 506903). α-γδ-PE antibody was purchased from BD Biosciences (Cat. 553178). If no intracellular staining was performed, DAPI (BioLegend, Cat. 422801) was added just before analysis on the cytometer to discriminate live and dead cells. For intracellular staining, Zombie Green (BioLegend, Cat. 423112) was used as a viability dye before incubation with the surface markers.

The following surface markers were used for the T helper subsets (all BioLegend): α-CD4-PerCP (Cat. 100539), α-CD3-PB Cat. 100214), or α-CD3-APC (Cat. 100236). The following antibodies were used as intracellular markers: α-IFNγ-PE/Cy7 (BioLegend, Cat. 505825), α-FoxP3-APC (Invitrogen, Cat. 17-5773-80), α-GATA3-PE (Invitrogen, Cat. 12-9966-42), and α-RORγT-PE (BD Biosciences, Cat. 562607). The viability dye eFluor780 was used during surface staining (Invitrogen, Cat. 65-0865-14) to discriminate live and dead cells in the T helper cell subsets.

The following antibodies were used to stain BMDCs: α-MHCII (I-A/I-E)-eFl450 (eBioscience, Cat. 48-5321), α-CD11c-PeCy7 (BioLegend, Cat. 117318), α-CD40-APC (BD Pharmingen, Cat. 558695), α-CD86-FITC (BD Pharmingen, Cat. 553691). For BMDMs, the following antibodies were additionally used: α-CD11b-PeCy7 (BioLegend, Cat. 101216), α-F4/80-FITC (BioLegend, Cat. 123108), α-CD80-PerCP (BD Pharmingen, Cat. 560526), α-CD86-PE (BioLegend, Cat. 105008). During surface staining, the viability dye eFluor780 (Invitrogen, Cat. 65-0865-14) was used to stain and gate out dead BMDC/BMDM. OTII-transgenic T-cells were identified using α-CD4-BV510 (BioLegend, Cat. 100559), α-FoxP3-APC (eBioscience, Cat. 17-5773-80), and the viability dye eFluor780 (Invitrogen, Cat. 65-0865-14). To track proliferation, T-cells were stained using the CellTrace Violet Cell Proliferation Kit (Invitrogen, Cat. C34557).

**Gene expression analysis**

The isolation of total RNA and its subsequent transcription into cDNA has previously been described [6]. In brief, total RNA was isolated using Qiazol, followed by a DNase I digestion, and subsequent reverse transcription into cDNA. For transcription, 4 µg total RNA was reverse transcribed from tissue using Oligo dt primers (Thermo Fisher, Cat. SO132) for 1 hour at 37°C, and 1 µg total RNA from cell culture experiments was reverse transcribed using random primers (Thermo Fisher, Cat. SO142) for 1 hour at 42°C. To be able to specifically detect murine *H2afj* mRNA levels, total RNA was reverse transcribed using an *H2afj-specific* primer: 5´-GCTCTTCACCTTCTGG-3´. Performance and analysis of the gene expression data were conducted as previously described [7]. Sequences of the self-designed primers can be found in Table S2. To calculate the relative mRNA values, the Ct values of the genes of interest were normalized to the Ct values of the reference gene (using the 2-ΔΔC_T_ method). *Actb* or *Rpl37a* was used as a reference gene for all presented gene expression data.

**Immunofluorescence staining**

Slides were rinsed for 2 min with PBS, dried, and tissue was permeabilized with 0.5% Triton X-100 in PBS for 10 min at 37 °C, followed by blocking with 4% normal donkey serum (NDS) in 0.5% Triton X-100 for 1 h at RT. Slides were rinsed with PBS for 3 × 5 min followed by incubation with α-CD45-PE (BioLegend, Cat. 103106) and α-H2A.J (antibodies-online, Cat. ABIN6971761) in 2 % NDS at 4 °C overnight. The next day, the tissue was rinsed for 3 × 5 min with PBS, incubated with α-rabbit secondary antibody (Jackson ImmunoResearch, Cat. 711-605-152), and covered with Fluoroshield + DAPI. Sections were dried at RT for 2 hours and stored at 4 °C, protected from light. Image acquisition was performed using a Keyence BZ-X microscope with DAPI, GFP, TRITC, and Cy5 filters. For IL17RA immunofluorescence staining, murine keratinocytes were fixed with 4% PFA in PBS for 30 min at room temperature, permeabilized with 0.5% Triton X-100 in PBS for 15 min at RT, followed by blocking with 10% FCS in PBS for 15 min at RT. The IL17RA antibody (1:300, Invitrogen, PA5101326) was incubated overnight at 4°C. Secondary antibody DyLight 488 anti-rabbit IgG (1:500, Invitrogen, 35552) was incubated for 45 min at RT in the dark. Nuclear staining was performed with DAPI (1:50.000) for 5 min at RT. Finally, the slides were covered with mounting medium (Fluoroshield™, Merck, F6182) and coverslips before image acquisition was performed using the Echo Revolve 4k microscope.

**Bulk RNA sequencing**

Both sequencing and library construction of total RNA were performed by Novogene (Munich, Germany). Sequencing of the quantified libraries was performed on the Illumina Novaseq X plus platform with paired-end 150-cycle (PE150) sequencing, according to effective library concentration and data amount. The sequencing raw reads were further processed by CLC Genomics Workbench (v25.0; Qiagen for T_H_17 and v24.0 for keratinocytes). The NGS core facility (Research Centre for Immunotherapy) performed mapping against the mouse reference genome GRCm38 for keratinocytes and GRCm39 for T_H_17 cells using CLC's default settings (mismatch cost = 2; insertion cost = 3; deletion cost = 3; length fraction = 0.8; similarity fraction = 0.8; global alignment = No; strand-specific = both; library type = bulk; maximum number of hits for a read = 10; count paired reads as two = no; ignore broken pairs = yes). Differentially expressed genes were defined as having a minimum absolute fold change > 2 with a difference cut-off of absolute > 4 and an adjusted p-value ≤ 0.05 between WT and *H2afj* KO. For the calculation of the p-value, a classical t-test (transcriptome analysis from keratinocytes) or a proportion test (Baggerley's test for transcriptome analysis from T_H_17) was conducted. The raw and processed data can be found under GEO accession numbers: GSE307885 (T_H_17) and GSE307884 (keratinocytes). The heatmaps were generated using Morpheus software (<https://software.broadinstitute.org/morpheus>). Gene set enrichment analysis was conducted using EnrichR for T_H_17 cells [8] or DAVID bioinformatics for keratinocytes [9].

**Single-cell sequencing analysis**

Public single-cell RNA-seq data of skin cells from healthy donors and psoriasis patients were downloaded from the Gene Expression Omnibus (Accession number: GSE173706) [10]. All analyses were performed using R V4.5.1. (R Core Team (2025). R: A language and environment for statistical computing. R Foundation for Statistical Computing, Vienna, Austria). Expression data were normalized using the NormalizeData function from Seurat 49. To identify likely doublets, we used scDblFinder (dbr.sd = 1) from the scDblFinder package (v1.22.0) and only retained cells that were classified as singlets in this analysis 50. Additionally, we removed cells with more than 40,000 unique molecular identifiers (UMIs), more than 15% mitochondrial transcripts, and a ratio between UMIs and detected genes above 10. We subsequently identified the 3.000 most variable features, scaled the expression values, and performed principal component analysis. A Uniform Manifold Approximation and Projection (UMAP) representation was derived using Seurat’s RunUMAP function and 10 principal components. We clustered cells by the Leiden algorithm (FindNeighbors and FindClusters(algorithm = 4) functions from Seurat), using 10 principal components and a resolution of 1.4. Based on the expression of published cell type markers, we identified clusters containing T-cells, basal keratinocytes, differentiated keratinocytes, dendritic cells or Langerhans cells, endothelial cells, smooth muscle cells or pericytes, melanocytes, Schwann cells, and fibroblasts. To identify FLGhigh LORhigh differentiated keratinocytes, we identified the 20 nearest neighbors for each cell with respect to 10 principal components. We then imputed expression of the genes FLG and LORICRIN by computing average normalized expression across the 20 nearest neighbors of each cell. Differentiated keratinocytes were classified as FLGhigh LORhigh if imputed expression values for both genes were more than one standard deviation above the respective mean across all differentiated keratinocytes.

**ELISA & Cytokine array**

To obtain samples for ELISA and cytokine array analysis, the skin samples were homogenized in protein lysis buffer (20 mM TRIS-HCl, pH 7.5; 150 mM NaCl; 1% Triton X-100; 1 mM Na_2_EDTA; 1 mM EGTA; 1 mM β-glycerophosphate; 2 M urea; and 1x protease inhibitor cocktail) in gentleMACS™ M Tubes on the gentleMACS™ Octo Dissociator, incubated for 10 min at 4°C, and then briefly sonicated for 10 min in a Bioruptor (Diagenode). Quantitative IL-17A protein detection from skin was performed using the ELISA MAX™ Deluxe Set Mouse IL-17A kit (BioLegend, Cat. 432504), according to the manual. The measurement was performed on a Hidex microplate reader (Hidex Sense), and the concentration was determined using a four-parameter logistic (4PL) regression.

The secretion of cytokines from the skin was performed as described in the manual of the Proteome Profiler Mouse Cytokine Array Kit Panel A (R&D Systems, Cat. ARY006). Before using the kit, an immunoblot analysis was performed to normalize the concentration of the samples, using β-Actin as a loading control (Cell Signaling, Cat. 3700). Afterwards, equal amounts of skin lysates derived from the skin of 3 different animals per group were mixed and applied to the membranes. All membranes were exposed at the same time. The relative expression of cytokines is shown as relative pixel values, retrieved using the dot plot analyzer in ImageJ. Relative pixel values were normalized to the average reference spots on the respective membranes.

**Table S1. ChIP and CUT&RUN expression primer.**

| **Primer** | **Forward primer (5´ - 3`)** | **Reverse Primer (5´ - 3`)** |
| --- | --- | --- |
| *Cxcl2* | CGCAGACATCACTTCCTTCC | AGCTGCCTGCCTCATTCTAC |
| *Ccl2* | TTGTCCATCTGGGTGTTAAAG | GCCGGAGAATCTGAAATGAC |
| *Cxcl5* | CAATTACAATAGCAGGGCTTCC | TGGGATTCTGCCAGCTTTG |
| *Il17a* | ATGAGTGCCGACAAACAACG | AGTGGGCTGCTGAATGACC |
| *Il17f* | CCCTGTTTCCACTGACCTCC | AGGGAGGGAAGATCTGGTCC |

Table listing all ChIP and CUT&RUN expression primers used in the manuscript.

**Table S2. Gene expression primer**

| **Primer** | **Forward primer (5´ - 3`)** | **Reverse Primer (5´ - 3`)** |
| --- | --- | --- |
| *Cxcl2* | CGCCCAGACAGAAGTCATAGC | CTTTGGTTCTTCCGTTGAGGG |
| *Ccl4* | AAGCCAGCTGTGGTATTCCTGAC | AACGTGAGGAGCAAGGACG |
| *Cxcl3* | ACCCAGACAGAAGTCATAGCC | GACACCGTTGGGATGGATCG |
| *Cxcl1* | ACGTGTTGACGCTTCCCTTG | TCCTTTGAACGTCTCTGTCCC |
| *Ccl21* | GATGACTCTGAGCCTCCTTAGC | CCGGGATGGGACAGCCTAAA |
| *Il1b* | AGCTGAAAGCTCTCCACCTC | GCTTGGGATCCACACTCTCC |
| *Il17f* | TGCTACTGTTGATGTTGGGAC | AATGCCCTGGTTTTGGTTGAA |
| *Il23a* | CAGCTCTCTCGGAATCTCTGC | TGTCCTTGAGTCCTTGTGGG |
| *Nfkbiz* | AACTCGCCAAGAGACCAGTG | AGAGCCACTGACTTGGAACG |
| *Il1a* | CTCATTGGCGCTTGAGTCGG | AGAGAGAGATGGTCAATGGCAG |
| *Ifng* | GACAATCAGGCCATCAGCAAC | CATTGAATGCTTGGCGCTGG |
| *Il4* | CTCACAGCAACGAAGAACACC | CCTTGGAAGCCCTACAGACG |
| *Il13* | AACATCACACAAGACCAGACTCC | CCAGGTCCACACTCCATACC |
| *Il17a* | GCCCTCAGACTACCTCAACC | TTCCCTCCGCATTGACACAG |
| *Il10* | GCATTTGAATTCCCTGGGTGAG | CATGGCCTTGTAGACACCTTGG |
| *Tgfb* | CCCGAAGCGGACTACTATGC | CATAGATGGCGTTGTTGCGG |
| *H2afj* | TCCGGGCGAGGCAAGCAG | CCGCAGCAGCCGGTGAAC |
| *Il6* | GTCCGGAGAGGAGACTTCAC | GCAAGTCGATCATCGTTGTTC |
| *Tnfa* | AAGTTCCCAAATGGCCTCCC | TTGCTACGACGTGGGCTAC |
| *Il12a* | ACCCTTGCATCTGGCGTCTAC | TTTAGGAGGGCAAGGGTGGC |
| *Cxcl5* | CCCTACGGTGGAAGTCATAGC | GAACACTGGCCGTTCTTTCC |
| *Ccl2* | CTGGAGCATCCACGTGTTGG | CCCATTCCTTCTTGGGGTCAG |
| *S100a9* | AATGGTGGAAGCACAGTTGG | CTGGTTTGTGTCCAGGTCCTC |
| *Ccl22* | AGGCTCAGCAAGCCCTATTC | CTCACAGAGTGACAGCCCAG |
| *Ahr* | CCAGGACCAGTGTAGAGCAC | AGCCATTCAGCGCCTGTAAC |
| *Il36g* | GTCAGCGTGACTATCCTCCC | TGGCTTCATTGGCTCAGGG |
| *Defb4* | GGTGCTGCTGTCTCCACTTG | TATTCATCTTGCTGGTTCTTCGTC |
| *S100a8* | TCGAGGAGTTCCTTGCGATG | TGCTACTCCTTGTGGCTGTC |
| *Ccl3* | TTCTGCTGACAAGCTCACCC | TCAACGATGAATTGGCGTGG |
| *Cxcl9* | GAGCAGTGTGGAGTTCGAGG | GGCAGGTTTGATCTCCGTTC |
| *Il17ra* | TCTTGGCCCAGGAACAATGG | CGGCCAAAGTTGTCTGATTCC |
| *Il17rc* | TGTGGACATGCGCCTCATAG | GCAGGTTGTTGGTCATGCAG |
| *Actb* | AGGAGTACGATGAGTCCGGC | GGTGTAAAACGCAGCTCAGTA |
| *Rpl37a* | CCAAGGCCTACCACCTTCAG | CTTGGGTTTCGGCGTTGTTC |

Table listing all gene expression primers used in the manuscript.

**References for the Supplementary Information**

1. Fischer B, Kubelbeck T, Kolb A, Ringen J, Waisman A, Wittmann M et al. IL-17A-driven psoriasis is critically dependent on IL-36 signaling. Front Immunol. 2023;14:1256133. doi:10.3389/fimmu.2023.1256133.

2. Zal T, Volkmann A, Stockinger B. Mechanisms of tolerance induction in major histocompatibility complex class II-restricted T cells specific for a blood-borne self-antigen. J Exp Med. 1994;180(6):2089-99. doi:10.1084/jem.180.6.2089.

3. Müller A, Dickmanns A, Resch C, Schäkel K, Hailfinger S, Dobbelstein M et al. The CDK4/6-EZH2 pathway is a potential therapeutic target for psoriasis. J Clin Invest. 2020;130(11):5765-81. doi:10.1172/JCI134217.

4. Bankhead P, Loughrey MB, Fernandez JA, Dombrowski Y, McArt DG, Dunne PD et al. QuPath: Open source software for digital pathology image analysis. Sci Rep. 2017;7(1):16878. doi:10.1038/s41598-017-17204-5.

5. Mendez J, Stillman B. Chromatin association of human origin recognition complex, cdc6, and minichromosome maintenance proteins during the cell cycle: assembly of prereplication complexes in late mitosis. Mol Cell Biol. 2000;20(22):8602-12. doi:10.1128/MCB.20.22.8602-8612.2000.

6. Müller A, Hennig A, Lorscheid S, Grondona P, Schulze-Osthoff K, Hailfinger S et al. IkappaBzeta is a key transcriptional regulator of IL-36-driven psoriasis-related gene expression in keratinocytes. Proc Natl Acad Sci U S A. 2018. doi:10.1073/pnas.1801377115.

7. Kolb A, Kulis-Mandic AM, Klein M, Stastny A, Haist M, Votteler V et al. Constitutive expression of the transcriptional co-activator IkappaBzeta promotes melanoma growth and immunotherapy resistance. Nat Commun. 2025;16(1):5387. doi:10.1038/s41467-025-60929-5.

8. Chen EY, Tan CM, Kou Y, Duan Q, Wang Z, Meirelles GV et al. Enrichr: interactive and collaborative HTML5 gene list enrichment analysis tool. BMC Bioinformatics. 2013;14:128. doi:10.1186/1471-2105-14-128.

9. Sherman BT, Hao M, Qiu J, Jiao X, Baseler MW, Lane HC et al. DAVID: a web server for functional enrichment analysis and functional annotation of gene lists (2021 update). Nucleic Acids Res. 2022;50(W1):W216-W21. doi:10.1093/nar/gkac194.

10. Ma F, Plazyo O, Billi AC, Tsoi LC, Xing X, Wasikowski R et al. Single cell and spatial sequencing define processes by which keratinocytes and fibroblasts amplify inflammatory responses in psoriasis. Nat Commun. 2023;14(1):3455. doi:10.1038/s41467-023-39020-4.
